# Mutator Suppression and Escape from Replication Error–Induced Extinction in Yeast

**DOI:** 10.1371/journal.pgen.1002282

**Published:** 2011-10-06

**Authors:** Alan J. Herr, Masanori Ogawa, Nicole A. Lawrence, Lindsey N. Williams, Julie M. Eggington, Mallika Singh, Robert A. Smith, Bradley D. Preston

**Affiliations:** Department of Pathology, University of Washington, Seattle, Washington, United States of America; The University of North Carolina at Chapel Hill, United States of America

## Abstract

Cells rely on a network of conserved pathways to govern DNA replication fidelity. Loss of polymerase proofreading or mismatch repair elevates spontaneous mutation and facilitates cellular adaptation. However, double mutants are inviable, suggesting that extreme mutation rates exceed an error threshold. Here we combine alleles that affect DNA polymerase δ (Pol δ) proofreading and mismatch repair to define the maximal error rate in haploid yeast and to characterize genetic suppressors of mutator phenotypes. We show that populations tolerate mutation rates 1,000-fold above wild-type levels but collapse when the rate exceeds 10^−3^ inactivating mutations per gene per cell division. Variants that escape this error-induced extinction (*eex*) rapidly emerge from mutator clones. One-third of the escape mutants result from second-site changes in Pol δ that suppress the proofreading-deficient phenotype, while two-thirds are extragenic. The structural locations of the Pol δ changes suggest multiple antimutator mechanisms. Our studies reveal the transient nature of eukaryotic mutators and show that mutator phenotypes are readily suppressed by genetic adaptation. This has implications for the role of mutator phenotypes in cancer.

## Author Summary

Organisms strike a balance between genetic continuity and change. Most cells are well adapted to their niches and therefore invest heavily in mechanisms that maintain accurate DNA replication. When cell populations are confronted with changing environmental conditions, “mutator” clones with high mutation rates emerge and readily adapt to the new conditions by rapidly acquiring beneficial mutations. However, deleterious mutations also accumulate, raising the question: what level of mutational burden can cell populations sustain before collapsing? Here we experimentally determine the maximal mutation rate in haploid yeast. We observe that yeast can withstand a 1,000-fold increase in mutation rate without losing colony forming capacity. Yet no strains survive a 10,000-fold increase in mutation rate. Escape mutants with an “anti-mutator” phenotype frequently emerge from cell populations undergoing this error-induced extinction. The diversity of antimutator changes suggests that strong mutator phenotypes in nature may be inherently transient, ensuring that rapid adaptation is followed by genetic attenuation which preserves the beneficial, adaptive mutations. These observations are relevant to microbial populations during infection as well as the somatic evolution of cancer cells.

## Introduction

Accurate DNA replication ensures the faithful transmission of genetic information between mother and daughter cells. To accomplish this important task, organisms have evolved a network of conserved pathways that govern DNA replication fidelity (reviewed in [1]). Polymerase proofreading and postreplication mismatch repair (MMR) are key determinants of fidelity, functioning to correct errors introduced by DNA polymerases during cell division (reviewed in [2]–[4]). Defects in these and other DNA repair pathways produce mutator phenotypes, which are characterized by increased rates of spontaneous mutation.

Mutator phenotypes arise spontaneously in nature and have mixed biological consequences (reviewed in [5]–[10]). In *Escherichia coli* and other bacteria, changing environmental conditions favor high mutation rates, which increase the likelihood of genetic adaptation [11]–[16]. However, after adaptation, mutator bacteria progressively lose fitness as they accumulate deleterious mutations in other genes [14], [17], and clones with lower mutation rates can evolve from mutator populations [14], [16], [18]–[20]. Thus, mutation rates in *E. coli* rise and fall as populations cycle through periods of adaptive and non-adaptive growth.

Mutators also impact eukaryotes. In mammals, mutator phenotypes fuel oncogenesis by providing the genetic diversity necessary for emergence of malignant clones [21], [22]. Many sporadic human tumors show signs of an elevated mutation rate [23], and inherited defects in polymerase proofreading [24]–[26] or MMR (reviewed in [27], [28]) confer mutator phenotypes and increase cancer risk. In the budding yeast *Saccharomyces cerevisiae*, loss of proofreading or MMR also elevates spontaneous mutation [29]–[34], and defective MMR can facilitate adaptation to changing environments [35], [36].

Similar to bacteria, eukaryotic mutator alleles are detrimental in the long-term. Deleterious mutations accumulate faster in mutator compared to non-mutator yeast strains [37], [38], and mutators eventually become extinct in a mutational meltdown process after serial passage through population bottlenecks [39]. This decline is accelerated in yeast with extreme mutation rates. Diploids that are homozygous defective for both proofreading and MMR grow slowly and have mutations rates that are elevated 10,000-times above wild-type levels [40]–[42]. Double-mutant spores germinate but arrest at various cell-cycle stages after 6–7 mitotic divisions [40], suggesting that the accumulation of DNA replication errors drives the extinction of haploid mutator strains.

Here, we experimentally define the threshold of error-induced extinction in haploid *S. cerevisiae* and show that cells readily escape extinction via genetic suppression. These escape mutants emerge rapidly and carry second-site mutations that suppress the mutator phenotype. Our findings show that mutators are intrinsically unstable and that spontaneous suppressors moderate high mutation rates in yeast.

## Results

### Abrupt Loss in Viability with Increased Mutation Rate

To obtain a range of mutator strains suitable for defining the maximal mutation rate in yeast, we conducted a mutagenesis screen of the *POL3* gene, which encodes the catalytic subunit of DNA polymerase δ (Pol δ). We used a plasmid shuffling strategy [29], [43] to introduce mutated *pol3* alleles into strains proficient (*MSH6*) or deficient (*msh6Δ*) for MMR (Figure S1). The screen focused on conserved residues in the proofreading exonuclease domain of Pol δ [44], [45] that, when mutated in *msh6Δ* cells, are expected to preferentially increase base-substitution and ±1 frameshift mutations [40], [46]–[49].

Our analysis identified 21 amino acid substitutions in the Pol δ proofreading domain that individually conferred a range of increased spontaneous mutation frequencies (Figure S2). These alleles had no observable effect on colony formation in *MSH6* cells. However, four alleles (*pol3-01*, *pol3-F406A, pol3-D407A* and *pol3*-*Y516F*) did not yield visible colonies when shuffled into *msh6Δ* cells. This result is consistent with previous reports of synthetic lethality between the proofreading-deficient *pol3-01* allele and MMR-defective alleles *pms1Δ*, *msh2Δ*, or *msh6Δ* [40], [41], [50].

**Figure 1:**
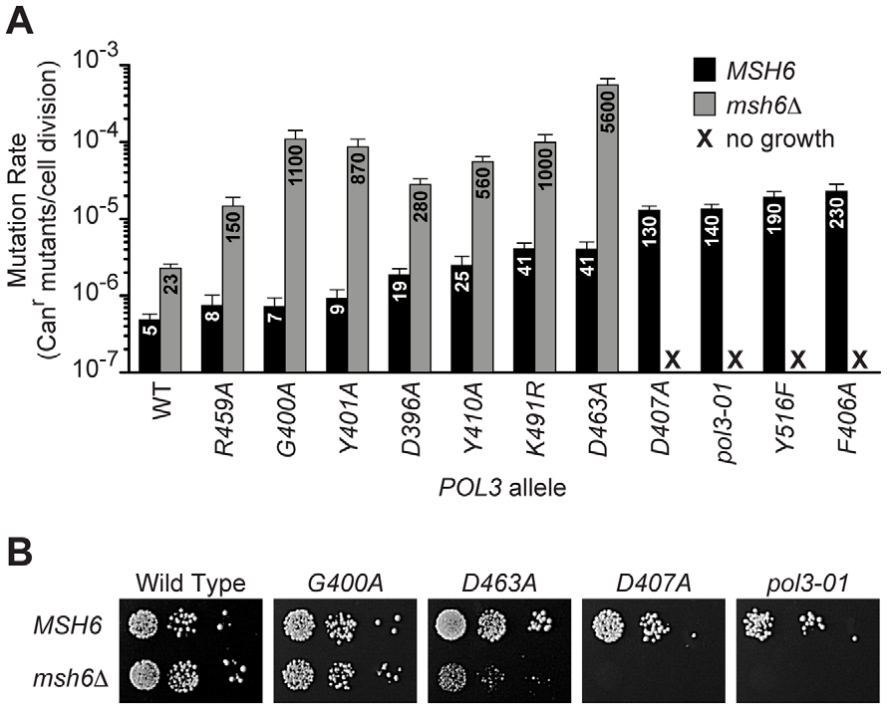
Evidence for a threshold of error-induced extinction. A) Entry into error-induced extinction. Mutated *pol3* alleles were introduced into haploid *MSH6* and *msh6Δ* yeast by plasmid shuffling (Figure S1), and mutation rates were measured by fluctuation assays and calculated using the maximum likelihood method. Each bar represents the spontaneous mutation rate, expressed as canavanine-resistant (Can^r^) mutants per cell division, conferred by a specific *POL3* allele in *MSH6* or *msh6Δ* cells. Mutation rate values (x 10^−7^) are indicated on each column. Error bars show 95% confidence intervals. WT, wild-type *POL3*; black, *MSH6*; gray, *msh6Δ*; **X**, no growth. B) Synthetic lethality of strong *pol3* mutator alleles with *msh6Δ*. Serial dilutions of haploid yeast containing *POL3–URA3* and *pol3–LEU2* plasmids were plated on SC FOA medium to select for cells that spontaneously lost *POL3–URA3*. Similar numbers of cells (∼10^5^, 10^4^ and 10^3^) were plated for each set of alleles in the *MSH6* and *msh6Δ* strains. Failed growth of *msh6Δ* cells carrying *pol3-D407A* or *pol3-01* indicates synthetic lethality (right two panels). *pol3-F406A* and *pol3-Y516F* also failed to support colony formation in *msh6Δ* cells (not shown). Note the small size of *pol3-D463A msh6Δ* colonies.

To determine whether the loss of growth capacity correlated with mutator strength, we quantified the spontaneous mutation rates of a subset of *pol3* alleles in the presence or absence of *MSH6* (Figure 1A). Alleles that imparted a 2- to 8-fold increase in the mutation rate of *MSH6* cells (*R459A*, *G400A*, *Y401A*, *D396A*, *Y410A*, *K491R* and *D463A*) were compatible with survival when *MSH6* was deleted. These *pol3 msh6Δ* double-mutants had mutation rates that were 15- to 150-times greater than the corresponding *pol3 MSH6* strains, consistent with synergy between *pol3* mutators and *msh6Δ*. In contrast, *pol3* alleles that increased the mutation rate 25- to 50-fold in *MSH6* cells (*D407A*, *pol3-01*, *Y516F* and *F406A*) conferred a loss of colony-forming capacity in *msh6Δ* cells (<1 colony/10^5^ cells plated; Figure 1B). Thus, the transition to no colony formation occurred over a narrow range of increasing mutation rates. This abrupt loss of growth capacity implies the existence of a threshold for error-induced extinction.

### Mutants That Escape Error-Induced Extinction

**Figure 2:**
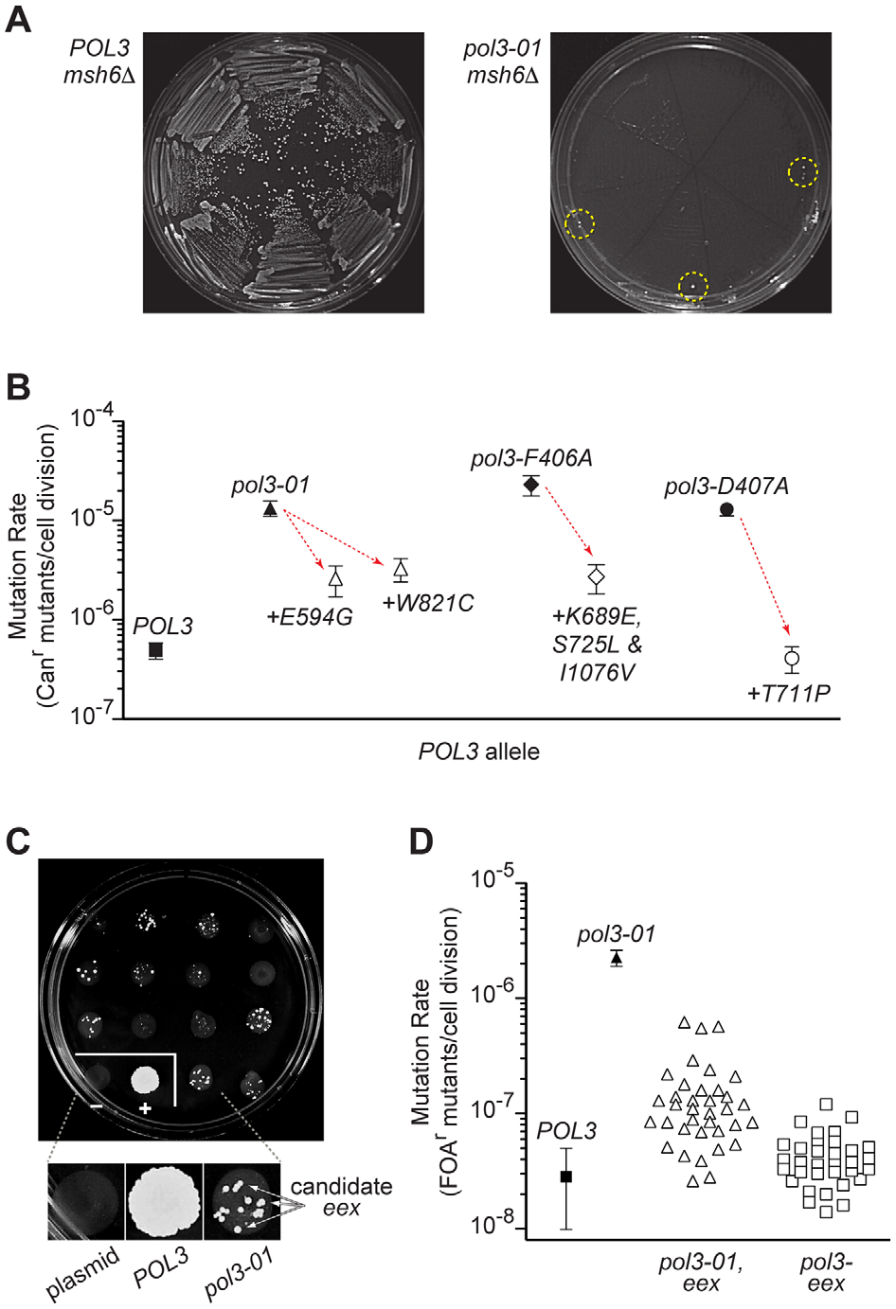
Escape from error-induced extinction. A) Emergence of colonies that escape *pol3-*01 *msh6Δ* synthetic lethality. Each segment of an FOA-containing SC plate (eight segments per plate) was streaked with an individual colony of *POL3–LEU2 POL3–URA3 msh6Δ* (left) or *pol3-*01*–LEU2 POL3–URA3 msh6Δ* (right) cells to select for loss of the *POL3–URA3* plasmid (see Figure S1). Resultant *POL3 msh6Δ* cells formed abundant visible colonies (left), whereas *pol3-01 msh6Δ* cells did not (right). Colonies that escape *pol3-01 msh6Δ* synthetic lethality (*eex* mutants) arose at low frequency near the outer margins of the plate (circled) where cell densities were highest. Similar results were seen when *pol3-F406A* or *pol3-D407A* were shuffled into *msh6Δ* cells (not shown). B) Antimutator effects of *eex* alleles encoding second-site changes in Pol δ. Rates of spontaneous mutation to canavanine-resistance (Can^r^) conferred by *pol3-01*, *pol3-F406A* or *pol-D407A* alone (filled symbols) and combined with intragenic *eex* alleles (open symbols) were determined in *MSH6* cells. Downward arrows illustrate the reduction in mutation rates (*i.e.*, the antimutator effect) caused by the second-site, amino-acid substitutions indicated beneath each datum point. Error bars show 95% confidence intervals. C) Representative plate from large-scale screen for *eex* mutants. Approximately 10^6^ cells from multiple independent *pol3-01–LEU POL3–URA msh6Δ* parent colonies were plated separately in ∼1-cm spots on FOA-containing SC medium. *LEU*-only and *POL3–LEU* plasmids were also shuffled into *msh6Δ* cells as controls. FOA-resistant colonies arose at varied frequencies from each parent clone. Insert, magnified view showing colonies that are candidate *eex* mutants. plasmid, LEU-only plasmid with no *POL3* gene. D) Mutation rates of *eex* mutants. Rates of spontaneous mutation to FOA-resistance (FOA^r^) were measured in a MMR-proficient strain with a chromosomal *URA3* reporter gene. Each datum point represents a different *POL3* allele. Mutation rates were determined from multiple independent fluctuation analyses of each allele. Confidence intervals (95%) are shown for *POL3* and *pol3-01*. Mutation rates and confidence intervals of individual *eex* alleles are in Table 1 and Table 2.

During our shuffling experiments, we observed occasional colonies that escaped *pol3 msh6Δ* synthetic lethality (Figure 2A). We speculated that second-site changes in Pol δ might rescue yeast from error-induced extinction by increasing DNA replication fidelity and thereby reducing the spontaneous mutation burden. To test this idea, we sequenced *pol3* plasmids from error-induced extinction *(eex*) mutants that escaped synthetic lethality between *msh6Δ* and *pol3-01*, *pol3-F406A* or *pol3-D407A*. The plasmids retained the original *pol3* mutator alleles, but also harbored additional second-site mutations in each *pol3* sequence. Our initial experiment yielded three *eex* mutants encoding single amino-acid substitutions in Pol δ (E594G or W821C in *pol3-01*; T711P in *pol3-D407A*) and two mutants with multiple substitutions (K689E, S725L and I1076V in *pol3-F406A*; R470C and T655A in *pol3-D407A*). Another mutation (*A894G*) was found in a large-colony variant of *pol3-D463A msh6Δ* cells. When the second-site *eex* mutations were re-engineered into new plasmids together with their corresponding mutator alleles (*pol3-01*, *pol3-*F406A or *pol3-*D407A), they rescued colony-forming capacity in *msh6Δ* cells and decreased the mutation rate of *MSH6* cells 10- to 33-fold (Figure 2B). The *eex* mutations appeared to be functionally interchangeable; *T711P* (the *pol3-D407A* suppressor) also rescued *pol3-01 msh6Δ* lethality, and either *T711P* or *E594G* (a *pol3-01* suppressor) restored normal growth to *pol3-D463A msh6Δ* cells. Considered together, these initial findings suggested that *eex* mutations within *POL3* confer escape from error-induced extinction by exerting an antimutator phenotype.

**Figure 3:**
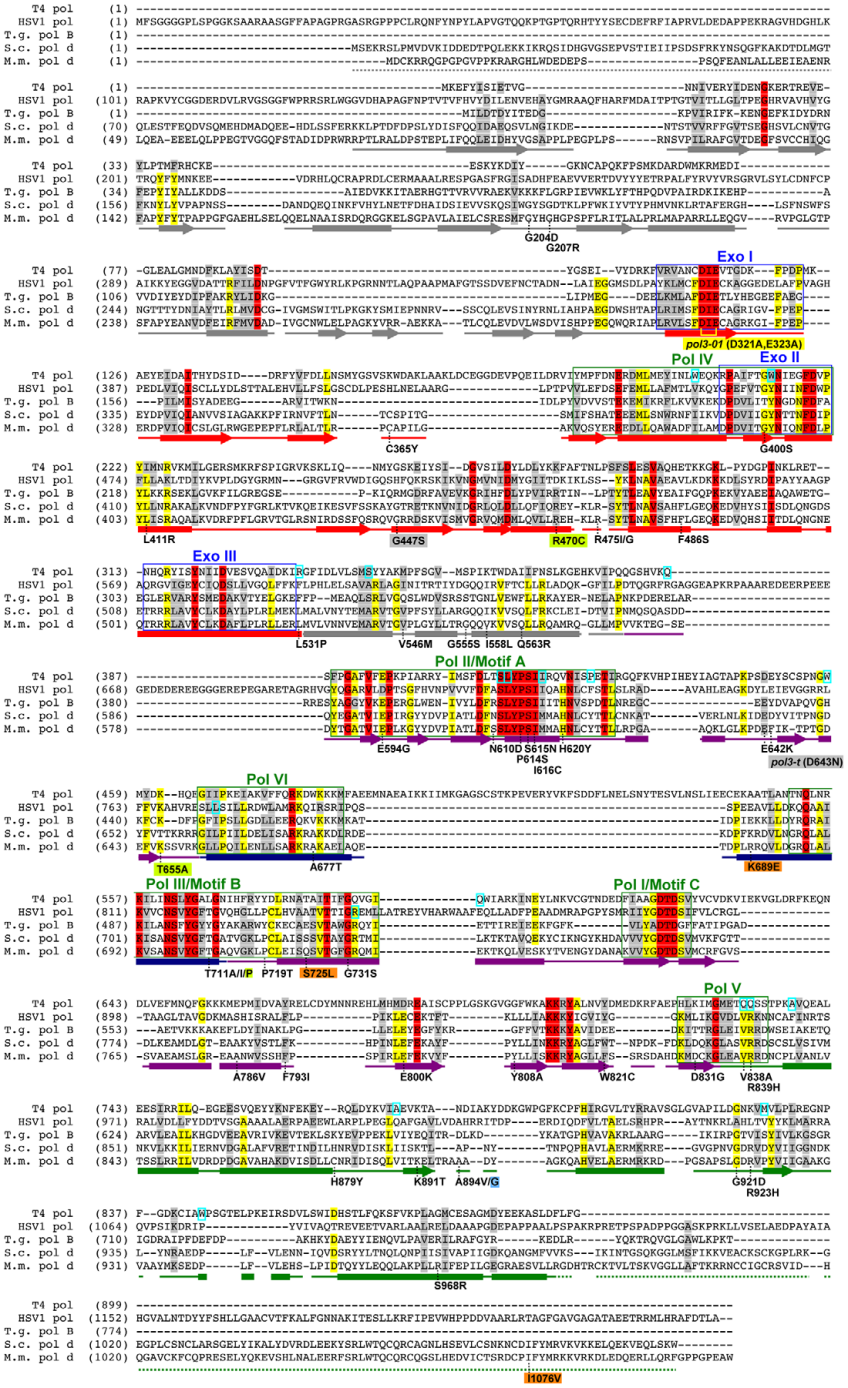
Amino-acid changes in Pol δ *eex* mutants. Aligned sequences of five B-family DNA polymerases: *Saccharomyces cerevisiae* Pol δ (S.c. pol d), *Mus musculus* Pol δ (M.m. pol d), *Thermococcus gorgonarius* (T.g. pol B), bacteriophage T4 (T4 pol), and herpes simplex virus 1 (HSV1 pol). Secondary structural elements of yeast Pol δ [74] are indicated below the alignment and color coded to depict their domain locations (see Figure 4A): rectangles, α-helices; arrows, β-strands; solid lines, loops; dotted lines, structure not solved. Conserved polymerase (Pol) and exonuclease (Exo) motifs are framed [44], [45], [120]. Amino-acid substitutions of interest in yeast Pol δ are placed underneath the alignment at the relevant positions, highlighted according to the following scheme: no highlight, *pol3-01,eex* mutations; green, *pol3-D407A,eex* mutations (R470C and T655A in one mutant, T711P in another); orange, *pol3-F406A,eex* mutations (three substitutions in the same mutant); blue, A894G mutation that rescued slow growth of *pol3-D463A msh6Δ* cells; yellow, *pol3-01* (D321A,E323A); gray, *pol3-t* (D643N) and G447S (previously identified antimutator alleles; [121], [122]). Residues that increase polymerase fidelity when mutated in T4 or HSV1 are indicated by aqua boxes in the alignment [4], [84]-[89], [123].

**Figure 4:**
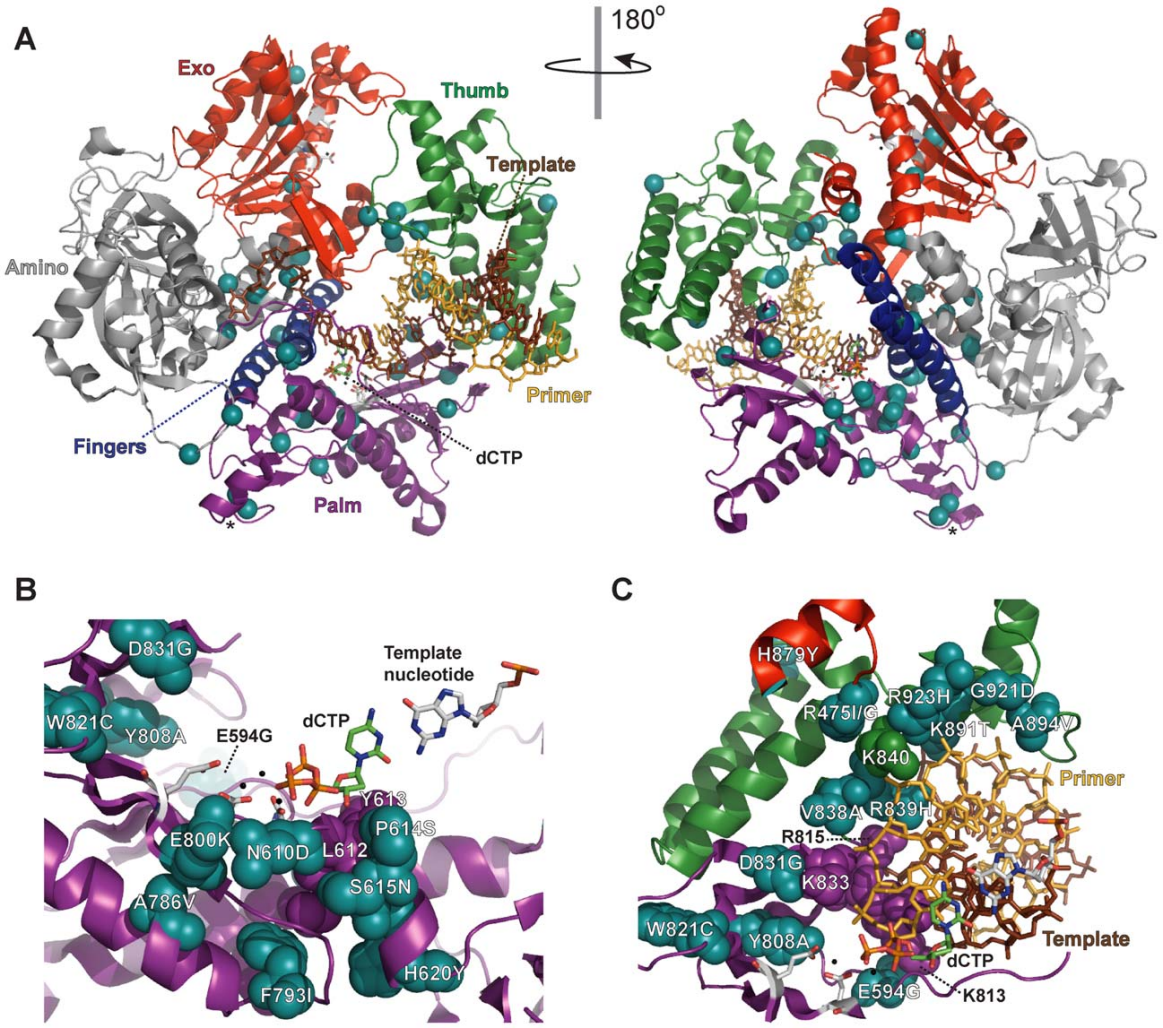
Locations of *eex* amino-acid substitutions in the *S. cerevisiae* Pol δ structure. A) Overall distribution of *eex* amino-acid substitutions. The catalytic subunit of yeast Pol δ is shown as a ribbon diagram with color-coded structural domains: amino, gray; exonuclease (Exo), red; palm, purple; fingers, blue; thumb, green. Other important elements are indicated as follows: DNA template strand, brown sticks; DNA primer strand, yellow sticks; incoming dCTP, green CPK sticks; metal ions, small black spheres; active-site residues, gray CPK sticks extending out from the α-carbon backbone in the palm and exonuclease domains. Residues changed by *eex* mutations are shown as light blue spheres. The asterisks mark adjacent E642K and D643N *eex* substitutions located on the solvent-exposed surface of Pol δ. Structure from [74] (Protein Data Bank accession code 3IAY). B) Amino-acid substitutions near the polymerase active site. Palm domain *eex* residues are shown as space-filling spheres (light blue) and labeled to indicate the amino-acid substitutions. Important non-mutated residues proximal to the *eex* substitutions are also shown as space-filling spheres (purple). The fingers and thumb domains have been removed for clarity. C) Amino-acid substitutions in the DNA binding track. View looking down the DNA helical axis. The primer (yellow) and template (brown) are held by a series of interactions along the DNA minor groove. *eex* residues are light blue. Amino acids positioned by *eex* residues and minor-groove ‘sensing’ residues in the palm domain (K813, K814 and R815; [74]) are shown as space-filling spheres colored according to domain as in panel (A). The three unpaired 5′ nucleotides of the template have been removed for clarity.

To obtain a broader view of escape mechanisms, we performed a large-scale screen for mutants that suppress the synthetic lethality between *pol3-01* and *msh6Δ* (Figure 2C). Mutants emerged from nearly every *pol3-01 msh6Δ* parent clone, and there was wide fluctuation in the number and size of mutant colonies, suggesting that escape variants arise randomly prior to selection on FOA. We isolated 113 independent *eex* mutants (Table S1). Seventy-four of these *eex* mutants carried *pol3-01* plasmids that still conferred lethality in a fresh *msh6Δ* strain. We infer that these mutants harbor mutations in chromosomal genes that influence DNA replication fidelity. The remaining 39 *eex* mutants carried *pol3-01* plasmids that did not cause synthetic lethality when isolated and independently re-shuffled into *msh6Δ* cells. DNA sequencing of these plasmids showed that, in addition to the *pol3-01* allele, each plasmid contained a different secondary mutation in *pol3*. These secondary mutations encoded single amino-acid changes in Pol δ (Figure 3 and Figure 4) and rescued colony-forming capacity when engineered *de novo* into *pol3-01* plasmids and shuffled into naïve *msh6Δ* cells. Consistent with our initial experiment, all of these intragenic *eex* mutations suppressed the *pol3-01* mutator phenotype, as measured at two different genetic loci (Figure 2D and Table 1). The weakest *eex* alleles suppressed mutation rates three-fold, while the strongest suppressors lowered mutation rates to wild-type levels (Figure 2D). Thus, cells escape *pol3-01 msh6Δ* lethality by acquiring any one of a variety of second-site mutations that suppress the mutator effect of Pol δ proofreading deficiency.

**Table 1:** Mutation Rates (×10^−7^) of *pol3-01,eex* Mutant Strains.

Allele		*MSH6* a				*msh6Δ* b		*msh6Δ* effect e
		FOA^r^		Can^r^		Can^r^		
*pol3-01* c		23	(19, 26)	135	(110, 157)	—		
+	*G207R*	6.2	(4.6, 8.1)	—		—		
+	*H879Y*	5.7	(4.5, 7.0)	53	(41, 65)	11000	(7700, 15000)	209
+	*Y808C*	5.5	(4.2, 7.0)	31	(25, 37)	7700	(6300, 9000)	198
+	*S968R*	2.9	(2.1, 3.8)	34	(26, 43)	5200	(4100, 6300)	153
+	*A894V*	2.4	(1.6, 3.5)	33	(25, 40)	3100	(2500, 3700)	96
+	*G400S*	2.2	(1.6, 3.0)	—		—		
+	*G204D*	2.1	(1.4, 2.9)	29	(22, 36)	4500	(3400, 5600) d	157
+	*L411R*	1.8	(1.0, 2.9)	—		—		
+	*E642K*	1.6	(1.1, 2.4)	23	(17, 28)	2600	(2000, 3000)	113
+	*F486S*	1.6	(1.0, 2.3)	—		—		
+	*T711A*	1.4	(1.0, 2.0)	33	(25, 41)	5900	(4700, 7100)	179
+	*R839H*	1.4	(0.9, 2.1)	—		—		
+	*W821C*	1.3	(0.8, 1.9	33	(24, 41)	2600	(2100, 3000)	80
+	*C365Y*	1.3	(0.8, 1.9)	—		—		
+	*A786V*	1.3	(0.8, 1.9)	12	(8, 15)	1600	(1200, 1900) d	135
+	*F793I*	1.2	(0.8, 1.9)	6	(4, 8)	1600	(1300, 1800)	258
+	*R475G*	1.2	(0.8, 1.8)	—		—		
+	*N610D*	1.1	(0.7, 1.6)	—		—		
+	*L531P*	1.0	(0.7, 1.6)	—		—		
+	*E594G*	1.0	(0.6, 1.6)	26	(17, 35) d	1700	(1200, 2100)	63
+	*R475I*	0.9	(0.5, 1.5)	14	(10, 18)	2700	(2300, 3200)	197
+	*D831G*	0.9	(0.5, 1.3)	11	(8, 15)	1700	(1400, 1900)	150
+	*G921D*	0.9	(0.4, 1.3)	—		—		
+	*D643N*	0.8	(0.4, 1.5)	—		—		
+	*V838A*	0.8	(0.5, 1.3)	—		—		
+	*R923H*	0.8	(0.4, 1.3)	—		—		
+	*E800K*	0.7	(0.4, 1.2)	13	(10, 17)	1700	(1400 1900)	126
+	*K891T*	0.7	(0.4, 1.2)	18	(14, 23)	1900	(1500 2300)	103
+	*H620Y*	0.7	(0.4, 1.1)	15	(12, 18)	3700	(3000, 4400)	248
+	*G731S*	0.5	(0.2, 1.0)	—		—		
+	*S615N*	0.5	(0.3, 0.9)	—		—		
+	*V546M*	0.5	(0.3, 0.8)	14	(10, 19)	2400	(1900, 2800)	171
+	*G555S*	0.4	(0.2, 0.8)	—		—		
+	*P614S*	0.4	(0.2, 0.8)	11	(8, 15)	2100	(1700, 2400)	185
+	*Q563R*	0.3	(0.1, 0.5)	13	(9, 17)	2000	(1500, 2500)	158
+	*I558L*	0.3	(0.1, 0.5)	—		—		

Rates of FOA or canavanine resistance (FOA^r^ or Can^r^ mutants per cell division) were determined by fluctuation analyses and maximum likelihood estimates using data from multiple independent experiments (except where noted). *pol3-01,eex* alleles are arranged from top to bottom according to the degree of mutator suppression as determined from rates of FOA resistance. Confidence intervals (95%) are in parentheses. A dash (–) indicates the mutation rate was not determined.

a Mutation rates were determined in two different strains: FOA^r^, BP4001; Can^r^, YP6.

b Mutation rates were determined in strain MP4.

c *pol3-01,eex* alleles not analyzed: *I616C*, *A677T*, and *P719T*.

d Mutation rate from a single experiment.

e The fold-increase in mutation rate caused by loss of MSH6 (*msh6Δ* effect) was calculated by dividing each *pol3-01,eex msh6Δ* mutation rate by the corresponding *pol3-01,eex MSH6* rate. On average, *msh6Δ* increased mutation rate 157-fold.

### Contributions of MMR and Proofreading to DNA Replication Fidelity *In Vivo*

The *eex* mutants provided an opportunity to assess the proportion of Pol δ errors that are repaired by proofreading and MMR *in vivo*. Taking advantage of the viability of *pol3-01,eex msh6Δ* cells, we first compared the mutation rates of isogenic strains that lack Pol δ proofreading and differ only in their MMR activity. The average increase in mutation rate in *pol3-01,eex* strains after deletion of *MSH6* was 157-fold (Table 1), consistent with Msh6-dependent repair of greater than 99% of the errors generated by proofreading-deficient Pol δ. As expected from the mutation biases conferred by *pol3-01* or *msh6Δ* alone [40], [46]–[48], spontaneous mutations in *pol3-01,eex msh6Δ* strains were almost exclusively base substitutions (Figure S3 and Table S2). Thus, our estimate primarily reflects the efficiency of base-base mismatch repair. This estimate is an average across multiple scoreable sites in *CAN1*; MMR efficiencies at individual sites may vary widely [51].

To similarly estimate the efficiency of Pol δ proofreading *in vivo*, we initially determined the influence of *eex* alleles on mutation rates in the presence of proofreading. Most MMR-proficient *pol3-eex* strains had no discernable mutator phenotype (Figure 2D, Table 2). However, in the absence of *MSH6* many of the *pol3-eex* alleles produced slightly higher mutation rates than the *POL3 msh6Δ* control (Table 2). These alleles were excluded from our analysis, because their weak mutator phenotypes may result from altered partitioning or other defects that reduce proofreading efficiency [4], [52]. Eight *pol3-eex msh6Δ* strains exhibited mutation rates within two-fold of *POL3 msh6Δ: G204D, H620Y*, *T711A*, *E594G*, *Y808C*, *W821C*, *H879Y*, and *S968R*. The mutation rates of these *pol3-eex msh6Δ* strains were compared to rates of the corresponding *pol3-01,eex msh6Δ* cells. This strategy allowed us to examine isogenic strains that differ only in their Pol δ proofreading activity and lack the masking effects of Msh6-mediated MMR. Proofreading deficiency increased mutation rates an average of 163-fold, indicating that the Pol δ exonuclease corrects greater than 99% of polymerase errors across the *CAN1* reporter gene. Assuming Pol δ proofreading and Msh6-dependent MMR act in series [40], we estimate their combined contribution to DNA replication fidelity in yeast at greater than 10^4^. Proofreading and MMR contribute similarly to replication fidelity in bacteria [53].

**Table 2:** Mutation Rates (×10^−7^) of *pol3-eex* Mutant Strains.

Allele		*MSH6* a				*msh6Δ* b		*pol3-01* effect e
		FOA^r^		Can^r^		Can^r^		
*POL3* c		0.3	(0.1, 0.5)	4.9	(4.0, 5.8)	23	(20, 26)	
+	*G207R*	0.4	(0.2, 0.7)	—		—		
+	*H879Y*	0.4	(0.2, 0.6)	1.9	(1.4, 2.6)	56	(44, 67)	198*
+	*Y808C*	0.4	(0.2, 0.7)	2.1	(1.4, 2.9)	39	(30, 48)	198*
+	*S968R*	0.3	(0.1, 0.5)	2.2	(1.6, 2.8) d	23	(17, 30)	226*
+	*A894V*	0.6	(0.3, 0.9)	2.7	(2.0, 3.4)	110	(88, 130)	29
+	*G400S*	1.2	(0.8, 1.8)	—		—		
+	*G204D*	0.7	(0.4, 1.1)	2.6	(1.9, 3.4)	54	(39, 70)	84*
+	*E642K*	0.5	(0.2, 1.0)	7.0	(5.6, 8.4)	150	(120, 180)	17
+	*F486S*	0.9	(0.6, 1.4)	—		—		
+	*T711A*	0.3	(0.5, 0.6)	2.1	(1.7, 2.4)	22	(15, 39)	275*
+	*R839H*	0.5	(0.2, 0.9)	—		—		
+	*W821C*	0.3	(0.1, 0.6)	3.0	(2.3, 3.8)	33	(27, 38)	78*
+	*C365Y*	0.3	(0.2, 0.5)	—		—		
+	*A786V*	0.1	(0.0, 0.3)	6.1	(4.3, 7.9) d	80	(63, 98)	19
+	*F793I*	0.2	(0.1, 0.4)	2.8	(2.0, 3.6) d	89	(71, 110)	179
+	*R475G*	0.2	(0.1, 0.4)	—		—		
+	*N610D*	0.5	(0.2, 0.9)	—		—		
+	*E594G*	0.2	(0.1, 0.5)	8.3	(5.5, 11.2) d	15	(12, 18)	107*
+	*R475I*	0.4	(0.2, 0.8)	5.8	(3.6, 8.3) d	160	(130, 180)	18
+	*D831G*	0.9	(0.5, 1.4)	2.9	(2.1, 3.7)	64	(50, 78)	27
+	*G921D*	0.4	(0.1, 0.8)	—		—		
+	*V838A*	0.5	(0.3, 0.9)	—		—		
+	*R923H*	0.2	(0.0, 0.4)	—		—		
+	*E800K*	0.7	(0.4, 1.2)	3.6	(2.7, 4.5)	120	(97, 160)	14
+	*K891T*	0.3	(0.1, 0.5)	6.6	(4.8, 8.3) d	220	(180, 260)	9
+	*H620Y*	0.3	(0.1, 0.6)	4.5	(2.8, 6.5) d	26	(19, 34)	141*
+	*G731S*	0.2	(0.1, 0.5)	—		—		
+	*S615N*	0.3	(0.1, 0.7)	—		—		
+	*V546M*	0.4	(0.2, 0.7)	4.3	(2.5, 6.5) d	71	(49, 93) d	33
+	*G555S*	0.6	(0.3, 1.0)	—		—		
+	*P614S*	0.5	(0.3, 1.0)	4.4	(3.3, 5.6)	130	(100, 160)	16
+	*Q563R*	0.3	(0.1, 0.6)	4.3	(3.0, 5.7)	120	(98, 140)	17
+	*I558L*	0.5	(0.2, 0.8)	—		—		

Rates of FOA or canavanine resistance (FOA^r^ or Can^r^ mutants per cell division) were determined by fluctuation analyses and maximum likelihood estimates using data from multiple independent experiments (except where noted). *eex* alleles are arranged from top to bottom in the same order as Table 1. Confidence intervals (95%) are in parentheses. A dash (–) indicates the mutation rate was not determined.

a Mutation rates were determined in two different strains: FOA^r^, BP4001; Can^r^, YP6.

b Mutation rates were determined in strain MP4.

c *pol3-eex* alleles not analyzed: *L411R*, *L531P*, *I616C*, *D643N*, *A677T*, and *P719T*.

d Mutation rate from a single experiment.

e The fold-increase in mutation rate caused by loss of Pol δ proofreading (*pol3-01* effect) was calculated by dividing each *pol3-01,eex msh6Δ* mutation rate (Table 1) by the corresponding *pol3,eex msh6Δ* rate (above). *pol3,eex msh6Δ* strains marked with an asterisk (*) have mutation rates within 2-fold of the *POL3 msh6Δ* strain, suggesting the *eex* alleles do not hamper primer partitioning or exonuclease function. In these strains, *pol3-01* increased mutation rate an average of 163-fold.

### Defining the Threshold of Error-Induced Extinction

**Figure 5:**
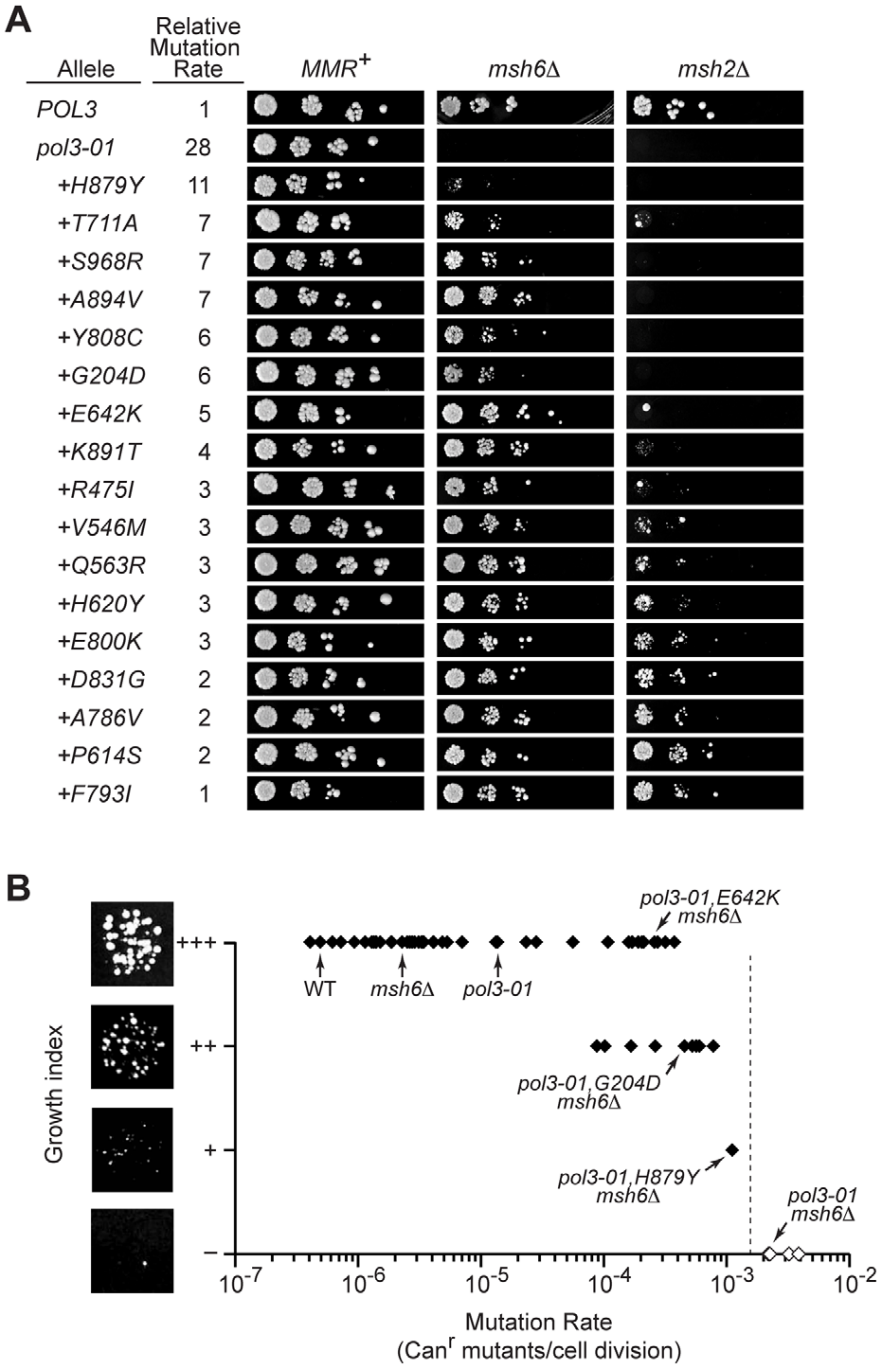
Defining the threshold of error-induced extinction. A) Plasmid shuffling experiments to reveal synthetic interactions between *pol3-01,eex* alleles and MMR mutations. Ten-fold serial dilutions of yeast containing *POL3–URA3* and *pol3–LEU2* plasmids were plated on FOA-containing SC medium to select for cells that spontaneously lost *POL3–URA3*. Similar numbers of colony forming units were plated for each set of alleles in the *MMR^+^*, *msh6Δ* and *msh2Δ* strains. Failed growth indicates synthetic lethality. Small colonies reflect slow-growth phenotypes. Relative mutation rates are the Can^r^ mutation rates conferred by each *pol3* allele relative to wild-type *POL3* in MMR-proficient cells (see Table 1 and Table 2). Alleles are listed in decreasing order of mutator strengths. Some alleles with statistically similar mutation rates (as reflected by overlapping confidence intervals) have slightly different relative rates due to mathematical round-off. B) Relationship between growth capacity and *CAN1* mutation rate for 62 haploid yeast strains. Colonies of *pol3-01,E642K msh6Δ*, *pol3-01,G204D msh6Δ*, *pol3-01,H879Y msh6Δ*, and *pol3-01 msh6Δ* cells are shown to illustrate wild-type (+++), moderately defective (++), severely defective (+), and failed (–) growth, respectively. The vertical dashed line indicates the estimated maximal mutation rate compatible with haploid yeast colony formation, which is our functional definition of the replication error threshold. Filled symbols, rates measured by fluctuation analyses. Open symbols, rates estimated as described in the text. Data in brackets with an asterisk (*) are *pol3-01,T711A msh6Δ*, *pol3-01,S968R msh6Δ*, *pol3-01,G204D msh6Δ*, and *pol3-01,Y808C msh6Δ*.

The *pol3-01,eex* MMR-proficient strains formed colonies with similar size and efficiency as the *POL3* control (Figure 5A, left). Thus, the corresponding *eex* mutant polymerases must suffice for the essential functions of Pol δ in replication [54]. However, in the absence of *MSH6*, *pol3-01,eex* alleles with the strongest mutator phenotypes impaired growth (Figure 5A, center). We used these mutants, together with synthetic-lethal alleles, to estimate the maximal mutation rate compatible with haploid yeast proliferation.

The upper and lower limits of the maximal rate were determined as follows. First, we calculated the predicted mutation rates of *msh6Δ* strains harboring synthetically lethal *pol3* alleles (*pol3-01*, *pol3-F406A*, *pol3-D407A* or *pol3-Y516F*) as the mutation rate of each *pol3 MSH6* strain (Figure 1A) times 157 (the average increase in rate observed upon deletion of *MSH6*; see preceding section). These predicted rates ranged from 2×10^−3^ Can^r^ mutants per cell division for *pol3-01 msh6Δ* and *pol3-D407A msh6Δ* to 4×10^-3^ for *pol3-F406A msh6Δ* (Figure 5B). Second, we determined the growth capacities of all mutator and suppressor strains in our collection using a semi-quantitative scale based on colony size. Wild-type colony-forming capacity (+++) was consistently observed at rates as high as 5×10^−5^ Can^r^ mutants per cell division (Figure 5B). As the mutation rate exceeded 5×10^-5^, several strains exhibited a slow-growth phenotype (++), and a single strain (*pol3-01,H879Y msh6Δ*) showed a severe growth deficit (+) at a mutation rate of 1×10^−3^. These results demonstrate that the maximal mutation rate is reached when there are ∼10^−3^ inactivating mutations in *CAN1* per cell division (Figure 5B). Rates exceeding this maximum result in a failure to form visible colonies (*i.e.,* error-induced extinction).

If the observed decline in viability is due to an error threshold, additional mutation stress should exacerbate the growth defect. We introduced *pol3-01,eex* alleles into *msh2Δ* cells, which are defective in both Msh6- and Msh3-mediated MMR and thus have Can^r^ mutation rates that are 2- to 3-fold higher than *msh6Δ* cells [3], [41], [47], [48]. Colonies were observed only in *pol3-01 msh2Δ* strains with the strongest mutator suppressor alleles (Figure 5A, right). Collectively, these data suggest that *pol3-01 msh6Δ* cells with weak mutator suppressors are on the edge of error-induced extinction and that eliminating *MSH2* increases mutation rates beyond an extinction threshold. Although *pol3-01 msh2Δ* strains with strong mutator suppressors formed distinct colonies, these colonies were generally smaller and less uniform than the *POL3 msh2Δ* control. This variability in colony size suggests that viable *pol3-01,eex msh2Δ* cells quickly accumulate deleterious mutations that compromise replicative fitness. The observation that growth is impaired at mutation rates 10-times less than the 10^−3^ threshold (Figure 5B) suggests that accumulation of random mutations can impose a loss in fitness and shows that the growth capacity of haploid yeast declines even under conditions of non-lethal mutation burden.

## Discussion

Mutators accelerate microbial adaptation and mammalian oncogenesis. However, the fitness cost of increased mutation imposes indirect selection pressure to reduce mutation rates. This counter-selection will occur after adaptation to a stable environment where conditions no longer favor the genetic potential of mutators. One possible mechanism to reduce mutation rates is the acquisition of compensatory alleles at modifier loci that suppress the mutator phenotype.

In this study, we took advantage of synergies between Pol δ proofreading and MMR to titrate yeast mutation rates up to lethal levels and study the fate of mutators under strong counter-selection. We found that *msh6Δ* cells carrying hypomorphic proofreading alleles abruptly lose viability over a narrow range of increasing mutation rates (Figure 1). Thus, cell survival requires both proofreading and MMR to limit potentially lethal mutations introduced by Pol δ. Mutant clones that escape this error-induced extinction arose spontaneously (Figure 2), frequently due to second-site changes in Pol δ (Figure 3 and Figure 4) that conferred antimutator phenotypes (Figure 2 and Table 1). Using our collection of mutator and antimutator strains, we found that the maximum mutation rate compatible with haploid yeast survival corresponds to ∼10^-3^ inactivating mutations in *CAN1* per cell division (Figure 5). These studies provide evidence for an error threshold in yeast and demonstrate that genetic suppressors of error-prone replication spontaneously arise in eukaryotic mutator cells.

Below we consider error thresholds in relation to genetic complexity and mutational robustness, and we discuss potential mechanisms of mutator suppression.

### Error Thresholds, Genetic Complexity, and Mutational Robustness

We observed loss of growth capacity when the *CAN1* mutation rate exceeds ∼10^−3^ inactivating mutations per cell division (Figure 5). The yeast genome is comprised of ∼6000 genes (http://www.yeastgenome.org). Thus, a mutation rate of 10^−3^ corresponds to the random inactivation of ∼6 genes per cell per replication cycle (assuming *CAN1* is typical). On average, one of these six mutations will involve a gene required for haploid cell viability [55], [56]. Thus, there is a high probability that cells above the maximal mutation rate will acquire a lethal mutation after a few cell divisions. The restoration of cell growth *via* antimutator alleles (Figure 2) supports this hypothesis. Stalled DNA synthesis at nascent 3′ mispairs [57] and S-phase checkpoint signalling [58] could also contribute to growth arrest in strong mutators. However, it is not evident how MMR defects would exacerbate 3′ mispair extension by Pol δ, and simultaneous loss of proofreading and MMR does not halt growth specifically in S-phase [40]. Rather, proofreading/MMR double mutants arrest with varied cell morphologies [40] that resemble those observed in a systematic promoter-repression screen of essential genes [59] (Figure S4). Considered together, the evidence suggests that random mutations in essential genes are a primary cause of error-induced extinction. Synthetic cooperative interactions of non-lethal alleles will also contribute as cells accrue multiple mutations [60], [61]. In a similar manner, bacteria exhibit a replication error threshold that correlates with the number of indispensable genes [62], suggesting that maximal mutation rates can be used to estimate the genetic complexity of vital pathways in other organisms.

Error thresholds are also evident in diploids. Although diploid genomes generally buffer cells against the deleterious effects of mutation accumulation [63], haploinsufficient alleles still pose a significant threat to fitness. In a comprehensive library of diploid yeast heterozygotes, up to 20% of the hemizygous mutant strains exhibit reduced fitness during growth competition [64]. Observations of mutation meltdown in MMR-deficient cells [39] and lethality conferred by a hyper-mutator Pol δ variant [65] argue that diploid yeast are subject to an error threshold. The combined loss of Pol δ proofreading and MMR is also synthetically lethal in mice [25]. Similar to the situation in yeast [40], mouse cells defective for both proofreading and MMR are initially viable but arrest after a limited number of mitotic divisions [25]. This cessation of growth presumably results from an accumulation of mutations in genes required for cell propagation and embryo development.

**Figure 6:**
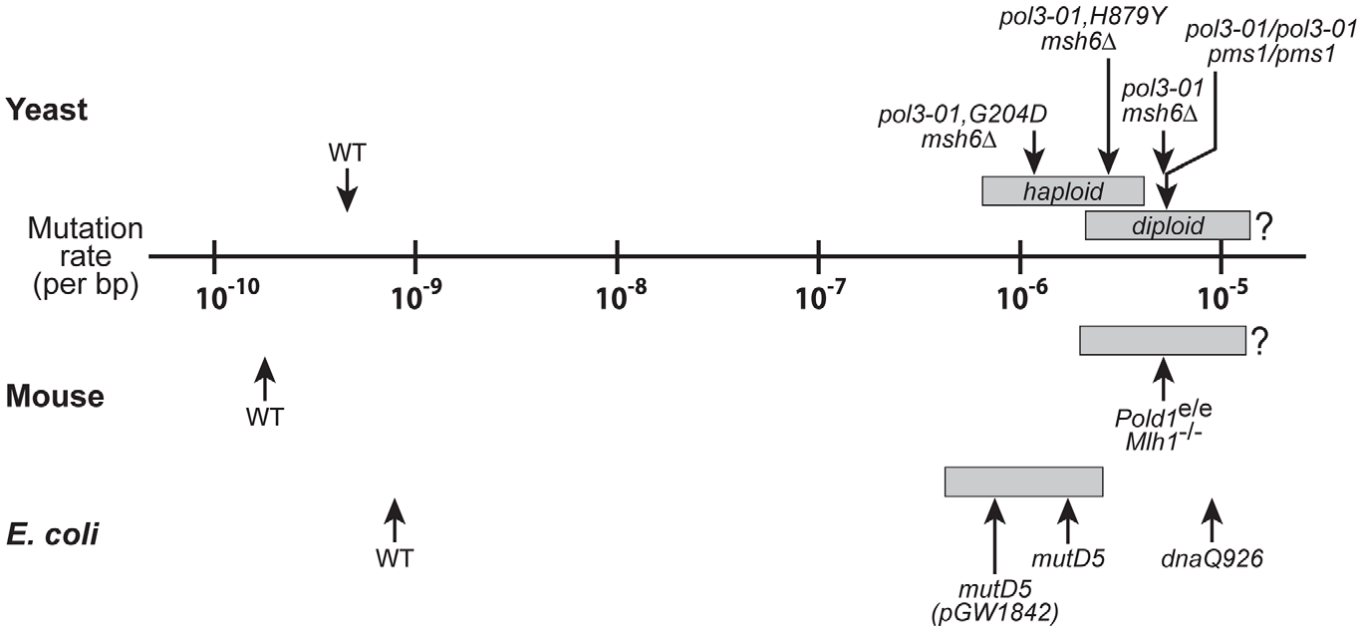
Mutational robustness of yeast, *E. coli*, and mice. Comparison of spontaneous per-base-pair mutation rates of wild-type (WT) and strong mutator strains of yeast (haploid and diploid), *E. coli* and mouse cells. Gray boxes indicate the mutation rate intervals that coincide with the transition from wild-type growth (leftmost boundary) to failed growth (rightmost boundary). The data for haploid yeast mutators are from Figure 5B, with the left boundary corresponding to the mutation rate of *pol3-01,E642K msh6Δ* cells (6.5×10^−7^) and the right boundary corresponding to the lethal threshold (4.1×10^−6^). The mutation rates of *pol3-01,G204D msh6Δ*, *pol3-01,H879Y msh6Δ*, and *pol3-01 msh6Δ* haploid yeast are shown as examples of progressively stronger mutators with slow (++), very slow (+) and no-growth (–) phenotypes, respectively. The data for *pol3-01/pol3-01 pms1/pms1* diploid yeast are from Morrison *et al.* [40]; *pol3-01/pol3-01 pms1/pms1* cells divide very slowly with a growth phenotype presumably in the range of + to ++. The mouse *Pold1^e/e^ Mlh1^Δ/Δ^* mutation rate is extrapolated from ouabain-resistance rates of cultured *Pold1^+/e^ Mlh1^Δ/Δ^* fibroblasts as described in Materials and Methods; a growth phenotype between + and ++ is assumed [25]. *E. coli* mutation rates and growth phenotypes are from Fijalkowska *et al.* [62]; *mutD5(pGW1842)*, *mutD5* and dnaQ926 exhibit slow (++), very slow (+) and no-growth (–) phenotypes, respectively. The positions of the gray boxes for diploid yeast, mouse and *E. coli* are estimates based on the mutation rate and growth capacity relationships observed in haploid yeast (Figure 5B). The error thresholds (rightmost boundaries) for diploid yeast and mouse cells are not known. The yeast wild-type rate is the average of multiple independent determinations (data herein and [40], [108], [114], [124]). Wild-type mouse and *E. coli* mutation rates are from Drake *et al.* [9], [114].

Although cells eventually succumb to error-induced extinction, they tolerate substantial increases in mutation rate before losing viability. This mutational robustness is apparent in yeast, *E. coli* and mouse cells (Figure 6). We show that haploid yeast tolerate more than a 1,000-fold increase in mutation rate before exhibiting overt loss of colony-forming capacity (Figure 5B), and a comparable increase in mutation rate is required to cause catastrophic errors in *E. coli* [62], suggesting that prokaryotes and haploid eukaryotes share similar degrees of robustness toward DNA polymerase errors. In comparison, diploid yeast and mouse cells retain replication capacity at mutation rates 10,000-times higher than wild-type levels (Figure 6; [25], [40], [66]). Thus, diploidy extends the threshold of error-induced cell death by five- to tenfold. These data suggest that cells can survive and persist during periods of high mutational loads. The maximal mutation rate will likely vary, depending on environmental conditions [67], [68], genetic redundancy [63], [69], [70], the plasticity of genetic interactions [71], [72], and the ability of cells to buffer deleterious changes in essential proteins [73].

### Structural Implications of the *eex* Amino-Acid Substitutions

We observed that escape mutants readily emerge when moderate mutators are pushed above the error threshold (Figure 2). One-third of the escape mutants resulted from second-site changes in Pol δ that suppress the proofreading-deficient mutator phenotype. Recent structural studies of *S. cerevisiae* Pol δ [74] provide insight into potential mechanisms of mutator suppression by these intragenic *eex* alleles (Figure 4, Figures S5 and S6).

Many *eex* mutations alter amino acids around the polymerase active site that are predicted to influence dNTP binding or catalysis (Figure 4B). Effects may be mediated by direct interactions of mutated residues with the metal•dNTP substrate or via packing interactions that indirectly affect the substrate binding pocket. Other *eex* mutations map to a stretch of amino acids that bind the template near the active site and buttress the fingers domain, which contains residues that contour the template•dNTP base pair (Figure S5). Amino-acid changes affecting active-site geometry, positioning of the template nucleotide, or stability of the catalytic conformation may act as antimutators by increasing selectivity for correct dNTPs or by slowing the rate of catalysis so that mispaired template•primers have more time to dissociate from Pol δ. A model of dissociation and subsequent editing by an alternative enzyme [20], [75] may best explain *eex* mutations that change amino acids along the DNA binding track (Figure 4C). Similarly, *eex* mutations in the exonuclease domain may impart structural changes that promote Pol δ dissociation during failed proofreading attempts (Figure S6). Intriguingly, two *eex* amino-acid substitutions (E642K and D643N) are located on the solvent-exposed surface of Pol δ (asterisk in Figure 4A), suggesting that changes in protein-protein interactions influence mutagenesis. Proteins encoded by *eex* loci extragenic to *POL3* (Table S1) are candidate interacting partners.

Several alternative enzymes may function to edit Pol δ errors in the *eex* mutants. One candidate is proofreading by Pol ε. Yeast with deficiencies in both Pol δ and Pol ε proofreading exhibit a synergistic increase in mutation rate, suggesting one or both polymerases may proofread for the other [46]. Other candidates include the 3′→5′ exonuclease activities of MRE11 [76] and Apn2 [77], or endonucleases such as Rad1/Rad10 or Mus81/Mms4 that cleave 3′ flap structures during replication fork restart [78]–[82]. An important consideration is that such alternative editing pathways may be redundant, with multiple activities masking the contributions of any one nuclease.

The locations of several *eex* substitutions in Pol δ resemble those of antimutators previously identified in bacteriophage T4 polymerase [4], [83]–[86] and in herpes simplex virus polymerase [87]–[89], two B-family DNA polymerases similar to Pol δ (Figure 3). Genetic screens have also identified *E. coli* DNA polymerase I and III antimutator variants, and similar to our findings, these *E. coli* antimutators result from diverse amino-acid substitutions throughout the polymerase structures [19], [20], [90]–[92]. Some amino-acid substitutions in T4 pol are thought to increase polymerase fidelity by promoting ‘hyper-editing’ of the primer terminus by the integral proofreading exonuclease (reviewed in [4]). However, the *eex* mutations we describe mediate their antimutator effects without the aid of an active exonuclease domain, similar to previously isolated *E. coli* antimutators [19], [20], [90].

Taken together, this structural analysis suggests two general antimutator mechanisms for Pol δ *eex* mutations: 1) increased dNTP discrimination, thereby making Pol δ more accurate, and 2) increased dissociation from mispaired primer-templates, thereby allowing other enzymes to proofread Pol δ errors. *eex* mutations could also decrease errors at Okazaki fragment junctions by suppressing the strand-displacement activity of proofreading-deficient Pol δ [52], [93]–[95].

### Evidence for Other Pathways of Mutator Suppression

Our study took advantage of synthetically lethal interactions between Pol δ proofreading and MMR alleles to select for antimutators. Several lines of evidence indicate that mutator suppressors also arise under non-lethal conditions and are not restricted to the Pol δ proofreading – MMR pathway. Morrison and Sugino observed mutator suppression in a yeast clone defective for Pol ε proofreading and MMR [46], and an engineered second-site mutation in Pol ε suppresses the mutator effect of Pol ε proofreading deficiency [96]. In *E. coli*, suppressors of diverse mutator pathways (MMR, proofreading and DNA damage repair) emerge spontaneously in strains that are well below the error threshold [14], [18]–[20]. In our studies, large-colony variants of slow-growing mutators were frequently observed (see, for example, Figure 5A), and in the one variant we pursued, we found the *A894G* suppressor mutation. Collectively, these studies show that many defects in DNA replication fidelity can be genetically suppressed and suggest that both moderate and strong mutators are intrinsically unstable.

The facile emergence of mutator suppressors that we observed in yeast suggests that similar pathways of suppression exist in multicellular eukaryotes. This has implications for the role of mutator phenotypes in cancer [22], [97]. During neoplastic transformation, mutator alleles that promote the formation of tumor cells are likely to incur a fitness cost due to an increase in mutational load. To offset this cost, suppressor alleles that reduce the mutation rate may emerge during the later stages of oncogenesis after genetic barriers to immortalization and metastasis have been overcome. Although recent findings suggest that a mutator phenotype persists in at least some types of human tumors [23], our results raise the prospect that mutator phenotypes may be transient during tumor progression due to genetic suppression. An analysis of mutation rate dynamics in cancer is warranted.

## Materials and Methods

### Media and Growth Conditions

 Yeast were grown at 30°C using YPD, synthetic complete (SC) media or SC drop-out media deficient in specific amino acids as needed to select for prototrophy [98]. Pre-formulated nutrient supplements for SC and SC lacking uracil and leucine were purchased from Bufferad. All other drop-out supplements were made as described [98]. *URA3*-deficient cells were selected on SC medium containing 1 mg/ml 5-fluroorotic-acid (FOA; Zymo Research) and an additional 50 mg/L uracil [43]. *TRP1*-deficient strains were selected on FAA selection media containing 0.5 mg/ml 5-fluroanthranillic acid (FAA) made as described [99]. Canavanine-resistant mutants were scored on SC plates lacking arginine that contained 60 µg/ml of canavanine. Reagents were obtained from Sigma-Aldrich or Fisher Scientific unless otherwise indicated.

### Yeast Plasmids and Strains

#### Plasmids

pGL310 is the *CEN4/ARS1/URA3* plasmid, YCp50 [100], modified to carry *SUP11* and the wild-type *POL3* gene under control of its native promoter [29], [101]. YCplac111*POL3* and YCplac111*pol3* derivatives are *CEN4/ARS1/LEU2* plasmids derived from YCplac111 [102] and contain the entire wild-type *POL3* or mutant *pol3* genes (with native promoters) flanked by HindIII and EcoRI restriction sites. pRS414*POL3* and pRS414*pol3-01* are derivatives of the CEN6/ARS4/TRP1 plasmid, pRS414 [103], carrying the HindIII-EcoRI *POL3* DNA fragments from YCplac111*POL3* and YCplac111*pol3-01*, respectively. The construction of YCplac111 and pRS414 vectors and subsequent subcloning of *eex* mutants are described in detail in Text S1.

#### Strains

Yeast strains and their genotypes are listed in Table S3. Chromosomal gene disruptions were introduced using PCR products generated with primers, templates, and protocols detailed in Table S4 and Text S1. YGL27-3D (a kind gift from Michel Simon and Gerard Faye, Institut Curie) is a haploid strain that carries a lethal, partial deletion of chromosomal *POL3* substituted by *HIS3* and complemented by pGL310. Chromosomal *MSH6* was replaced with *TRP1* to create YGL27-3Dmsh6dis4. To limit gene conversion between the *pol3* plasmids and residual *POL3* sequences in the chromosome, the entire *POL3* coding sequences in YGL27-3D and YGL27-3Dmsh6dis4 were replaced with *kanMX* [104], creating YP6 (previously called YGL27-*pol3Δ* [105]) and MP4, respectively.

The S288c derivative, BY4733 [103], was modified to create a set of isogenic strains in a standard genetic background. BY4733 was transformed with pGL310, and chromosomal *POL3* was deleted and replaced with *HIS3* to create P3H3a. *MSH2* was deleted in P3H3a and replaced with *TRP1* to create BP0109. *MSH6* was deleted in P3H3a and replaced with *kanMX* [106] to create BP1506. P3H3a was modified as follows to allow mutation rate measurements at *URA3*. First, P3H3a was transformed with pRS414*POL3* and plated on SC FOA to isolate a strain that lost pGL310. Then, to create BP4001, *AGP1* on Chromosome III was replaced with *URA3* oriented with the direction of transcription towards ARS306 [107].

### Plasmid Shuffling

Plasmid shuffling with pGL310-containing strains was carried out essentially as described [29], [43] (Figure S1). Cells transformed with YCplac111*pol3* plasmids, YCplac111*POL3* (positive control), or YCplac111 (negative control) were plated on SC lacking uracil and leucine. Cells transformed with pRS414*pol3-01*, pRS414*POL3* (positive control), or pRS414 (negative control) were plated on SC lacking uracil and tryptophan. After three days at 30°C, individual colonies were picked and resuspended in sterile H_2_O, and serial dilutions containing approximately 10^5^, 10^4^, 10^3^, or 10^2^ cells were plated onto SC or SC FOA to select for clones that spontaneously lost the *URA3* plasmid pGL310. A similar approach was used for shuffling in strains carrying the *TRP1* plasmid pRS414*POL3*; BP4001 transformants containing both pRS414*POL3* and YCplac111-based plasmids were selected on SC lacking tryptophan and leucine and then plated onto SC FAA to select for clones that spontaneously lost the *TRP1* plasmid.

### *eex* Mutant Screen

For the systematic isolation of spontaneous *pol3-01,eex* mutant alleles (Figure 2C), a *pol3-01* plasmid was transformed into *pol3Δ msh6Δ* + pGL310 yeast, and 1–5×10^6^ cells from independent transformants were plated separately on SC FOA. FOA-resistant clones were isolated in the MP4 strain carrying YCplac111*pol3-01* or in BP1506 carrying YCplac111*pol3-01* or pRS414*pol3-01*. *Bona fide eex* mutants were distinguished from FOA-resistant clones that result from *ura3* mutation or *pol3-01*→*POL3* gene conversion by using a genotyping assay described in Text S1. *pol3-01* plasmids from individual *eex* mutants were recovered and reshuffled into naïve *pol3Δ msh6Δ* + pGL310 cells to identify suppressors intragenic to *pol3-01*. Plasmids that conferred consistent survival upon reshuffling were purified, and the *pol3* genes were sequenced (primer sequences available on request). Intragenic *eex* alleles thus identified were individually re-engineered into fresh YCplac111*POL3* and YCplac111*pol3-01* vectors and re-transformed into MP4 or BP1506 stock strains as a final confirmation of the ability of each allele to confer the *eex* phenotype. The re-engineered mutants were used to assess the effects of *eex* alleles on mutation rates and plating efficiencies.

### Mutation Frequencies and Rates

For the scanning mutagenesis screen (Figure S2), sequence-verified YCplac111*pol3* plasmids were shuffled into YP6 or MP4 cells immediately prior to each experiment. Twelve to thirteen independent FOA-resistant colonies of each genotype were streaked onto SC plates in ∼1-cm patches, grown two days at 30°C, and then replica-plated to canavanine plates to qualitatively assess mutant frequencies based on the number of canavanine-resistant colonies [47] (Figure S2A).

To measure mutation rates at the *CAN1* locus, freshly streaked YP6 or MP4 strains were transformed with YCplac111*POL3* or YCplac111*pol3* plasmids, and multiple independent transformants were shuffled on SC FOA plates to obtain well-isolated single colonies. For each genotype, seven to eleven independent colonies, 1–2 mm in diameter, were excised as an agar plug, resuspended in 1 ml of dH_2_O, and sonicated briefly. To estimate the number of cell divisions (Nt) during colony formation, serial dilutions were plated on SC media, and the number of colony-forming units was counted after two days at 30°C. To determine the number of mutants for wild-type and weak mutator strains, all of the remaining cells were plated on canavanine plates; for stronger mutators, the cell suspension was diluted 1∶10 to 1∶200 in dH_2_0 prior to plating. The numbers of canavanine-resistant colonies on each plate were scored after three to four days at 30°C.

To measure *URA3* mutation rates, BP4001 was transformed with YCplac111*POL3,* YCplac111*pol3-01*, or their respective *eex* mutant derivatives. Four FAA-resistant colonies from independent transformants with each plasmid were inoculated into separate 100-µl SC overnight cultures. The following morning the cultures were diluted to 1000 cells/ml and, for each of the four isolates, 12 parallel 100-µl cultures (100 cells/culture) were set up in 96-well microtiter plates. The plates were sealed with adhesive PCR plate sealers (Abgene, AB-0558) to minimize evaporation [108]. After two days of growth at 30°C, the cells were re-suspended by vigorous vortexing, and nine of the replicate cultures were spot-plated in 200-µl volumes on SC FOA plates. To estimate the total number of cell divisions, the remaining three replica cultures from each isolate were combined, diluted, and plated on SC plates. Colony numbers were scored after 3–4 days. We confirmed that spot plating accurately determines the number of FOA-resistant colonies for the strongest mutator by dividing test cell suspensions in half and comparing colony counts in a 100-µl spot with 100 µl of the same suspension spread over an entire SC FOA plate.

Mutation rates were determined from the number of mutant colonies in each replica by calculating an estimate for *m* by maximum likelihood [109] using newtonLD in Salvador 2.1 [110] with Mathematica 6.0 (Wolfram Research) and dividing by the number of cell divisions inferred from colony forming units. Where values for Nt from independent experiments differed by less than 2-fold, the data sets were combined for the mutation rate calculations [109]. In some instances, Nt values from independent experiments differed by more than 2-fold. In most cases, the independently-derived mutation rates were similar and a single value was reported (noted in Table 1). Confidence intervals were calculated in Salvador 2.1 using LRIntervalLD, which relies on likelihood ratios [110].

From these mutation rates, the efficiency of Msh6-dependent MMR (*e_m6_*), expressed as the percentage of errors corrected, was calculated using equation 1:
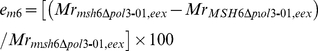
(1)where *Mr* is the relative mutation rate of the strain indicated in the subscript. The efficiency of Pol δ proofreading (*e_δexo_*), expressed as a percentage of errors corrected, was calculated similarly from equation 2:
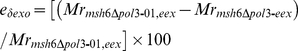
(2)


### *CAN1* Mutation Spectra

For each strain, we isolated up to 48 canavanine-resistant mutants from 48 independent shuffling experiments. Cells were treated with Zymolyase (ICN Biomedicals; 50 u/ml in 10 mM Tris•HCl/0.1 mM EDTA, pH7.5 at 37°C for 30 min then 95°C for 10 min), and the *can1* coding sequence was PCR-amplified in 50-µl reactions with Phusion polymerase (NEB) using primers can1F1N (5′-GGTTAAGATAAGTAGATAAGAGAATGATACG-3′) and can1S1 (5′-GCGTGGAAATGTGATCAAAGG-3′) with the following PCR conditions: 98°C, 1 min.; 35× (98°C, 10 sec.; 45°C, 30 sec.; 72°C, 90 sec.); 72°C, 1 min. The samples were treated with 5 units each of Antarctic phosphatase and Exo1 (New England Biolabs) to degrade excess primer and dNTPs, heated at 80°C for 20 min to inactivate the enzymes, and then sequenced with primers can1S1, can1S2 (5′-CCAAAGCGCCAAATGCAGCAG-3′), can1S3 (5′-TCCAATAACGGAATCCAACTG-3′) and can1S4 (5′-GGGCAATCATACCAATATGTC-3′). Mutation spectra were tabulated and compared using iMARS [111].

### Per-Base-Pair Mutation Rates

Phenotypic mutation rates were converted to per-base-pair rates using the approach of Drake [112]–[114] according to equations 3 – 5:
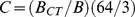
(3)




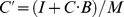
(4)




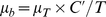
(5)
*C* and *C*′ ´ (equations 3 and 4) are correction factors to adjust for undetected (phenotypically silent) base-substitution mutations in a reporter gene. *B_CT_*  =  the number of chain-terminating base substitutions (3 possible codons), *B*  =  the number of all base substitutions (64 possible codons), and *I*  =  the number of insertions+deletions (indels) in representative mutation spectra from *M* mutants sequenced. The mutation rate per base pair (*μ_b_*) is calculated using equation 5 from the experimentally determined phenotypic mutation rate (*μ_T_*) multiplied by the correction factor *C*′ ´ and divided by the number of base pairs in the mutation-reporter target sequence (*T*). The effective target size (*τ*) is estimated by *T* / *C* ´′.

In our collection of 484 Can^r^ mutants from proofreading- and MMR-deficient yeast (Figure S3 and Table S2), there were 442 base substitutions (101 chain-terminating + 341 missense) and 42 indels (including infrequent complex mutations) in *CAN1* (*T* = 1773). Thus, *C* = 4.87 and *τ* = 391 base pairs. These values from mutator yeast strains are similar to those previously determined by others scoring spontaneous mutation in wild-type yeast (*C* = 4.73, [113]; *τ* = 236, [108]). The per-base-pair rates for haploid yeast plotted in Figure 6 were calculated from our Can^r^ *μ_T_* values (Table 1 and Table 2) with *C* = 4.87, *C*′ ´ = 4.53 and *T* = 1773. For diploid *pol3-01/pol3-01 pms1/pms1* yeast, we used the FOA^r^ mutation rate (*μ_T_*) of 3.5×10^-4^ reported by Morrison *et. al.* [40]; *C* = 8.18 (determined from the data of Lang and Murray [108]), *C*′ ´ = 6.79 and *T* = *804* base pairs for the *URA3* target gene. Thus, *τ* is 118 base pairs, and the per-base-pair mutation rate of *pol3-01/pol3-01 pms1/pms1* diploids at the *URA3* locus is [(3.5×10^−4^) ×6.79 / 804]  = 3.0×10^−6^. In Figure 6 we multiply this rate by 1.8 to adjust for the lower intrinsic mutation rate of *URA3* compared to *CAN1* (Table 1 and Table 2 and [108]).

For mouse cells, per-base-pair mutation rates were calculated from ouabain-resistance (Oua^r^) rates determined in our laboratory using spontaneously immortalized mouse embryo fibroblasts ([25] and unpublished data). The effective target size (*τ*) is estimated as follows. Base substitution mutations in any one of sixteen codons in the Na,K-ATPase α1 gene (*Atp1a1*) are known to confer genetically dominant resistance to µM concentrations of ouabain in human cells [115]. Mouse cells, however, are naturally resistant to µM concentrations of ouabain due to differences at 2 of these 16 codons (Q111R and N122D; [116], [117]. Our fluctuation assays were conducted with 2 mM ouabain [25], conditions expected to only detect mutations that confer exceptionally high ouabain resistance. We estimate the target size to be ∼5 base pairs per allele, corresponding to two *Atp1a1* codons (D121 and T797) known to effect >50-fold ouabain-resistance when mutated [115], [118]. Mouse fibroblast cell lines are typically tetraploid [119]. Therefore *τ* = 5 base pairs per allele×4 alleles = 20 base pairs. *Pold1^+/e^ Mlh1^Δ/Δ^* cells, which are heterozygous defective for Pol δ proofreading and nullizygous for MMR, exhibited a mutation rate of 65×10^−7^ Oua^r^ mutants per cell division (95% confidence interval = 56–75×10^−7^). This phenotypic rate corresponds to a per-base-pair rate of 65×10^−7^ / 20 base pairs  = 3.3×10^−7^. Mouse cells that are homozygous deficient for both Pol δ proofreading and MMR (*Pold1^e/e^ Mlh1^Δ/Δ^*) are viable but divide slowly up to embryonic day E9.5 [25]. Based on the relative mutation rates of *MMR^Δ/Δ^* diploid yeast with +/- or −/− Pol δ proofreading alleles [40], we estimate the per-base-pair rate of *Pold1^e/e^ Mlh1^Δ/Δ^* mouse cells to be 5×10^−6^.

## Supporting Information

Figure S1 Plasmid shuffling strategy. Mutated *pol3* alleles were introduced into *MSH6* and *msh6Δ* yeast by plasmid shuffling. Haploid yeast with a chromosomal deletion of *POL3* (*pol3Δ*) complemented by a wild-type *POL3*–*URA3* plasmid (left) are transformed with mutant *pol3–LEU2* plasmids. Individual colonies carrying both the *pol3* and *POL3* plasmids are isolated (center), and dispersed cells are then plated on 5-fluoroorotic acid (FOA) media to select mutant *pol3–LEU2* clones that lost the wild-type *POL3*–*URA3* vector (right).
(PDF)

Figure S2 Mutagenesis screen of the Pol δ exonuclease domain. Each allele was engineered into a wild-type *POL3* vector (YCplac111POL3) by site-directed mutagenesis and then introduced into yeast by plasmid shuffling (see Figure S1). Independent FOA-resistant colonies were patched onto synthetic complete (SC) plates and then replica-plated to SC plates lacking arginine and containing canavanine (60 µg/ml) to assess mutator phenotypes [47]. A) Representative canavanine plates used to assess mutator phenotypes. Each plate has twelve or thirteen patches of cells derived from independent colonies of the indicated genotypes. Spontaneous canavanine-resistant (Can^r^) mutants appear as small colonies in the ∼1-cm patches. Mutant frequencies were qualitatively judged from the lowest (wild type, WT) to highest (*pol3-G400A msh6Δ*) as indicated by –, +, ++ and +++ scores. B) Summary of *POL3* alleles and corresponding mutator and growth phenotypes in *MSH6* and *msh6Δ* cells.
(PDF)

Figure S3 Spontaneous *CAN1* mutations from *pol3-01,eex msh6Δ* cells. The *can1* coding sequences from 30–48 independent canavanine-resistant (Can^r^) mutants of each strain were PCR-amplified and sequenced. Spontaneous mutations identified in different strains are color coded according to the key at the bottom. Each base letter above the wild-type *CAN1* sequence indicates the site and nature of an independent base substitution or frameshift (+ or -) mutation. *CAN1* sequences involved in complex mutations are indicated by horizontal colored lines. Multiple mutations identified in *can1* from the same mutant clone are designated by the same superscript in the same color code. We observed mutation hotspots in *CAN1* that arose in multiple independent Can^r^ clones. One hotspot, a C to T mutation at nt 899, occurred in eight independent *pol3-01,K891T* Can^r^ clones. Two of the eight mutants had a second mutation elsewhere in the *CAN1* sequence, unambiguously identifying each clone as unique. This suggests that the abundance of mutations at this site is unlikely to be an artifact. One Can^r^ *POL3 msh6Δ* mutant (not shown) contained an insertion/deletion mutation that was evident by a larger PCR product; sequencing with nested *CAN1*-specific primers revealed an insertion containing the gene *RRP45.*
(PDF)

Figure S4 Similar cell morphologies in yeast after error extinction or repression of essential genes. Black bars: terminal cell morphologies of *pol3-01 pms1Δ* haploid cells that ceased growing due to error extinction [40]. White bars: cell-cycle arrest phenotypes of 563 haploid strains, each with a different repressed essential gene [59]. In the repression study, 82 additional essential genes showed a growth defect but with no defined cell-cycle arrest phenotype.
(PDF)

Figure S5 *eex* amino-acid substitutions near the template nucleotide in Pol δ. Schematic of the α-carbon backbone of Pol δ with residues of interest depicted as space-filling spheres. Structural elements are color-coded as in Figure 4 with the template•dNTP (T_0_P_0_) and polymerase active-site residues shown as CPK sticks. Amino acids changed by *eex* mutations are shown as light blue spheres and labeled to indicate the *eex* substitutions. Residues V546, G555, I558, and Q563 from the amino domain are in three closely associated α-helices that bind the template and buttress the fingers domain. The exo domain has been removed for clarity, and the penultimate T_1_P_1_ base-pair (brown and gold spheres) is included to delineate the binding pocket. Panel (A) is a view looking down on the DNA major groove. Panel (B) is the same image rotated 90° around the x-axis. The T_1_P_1_ base-pair was removed in Panel (B) to reveal positions of amino-acid substitutions around the template•dNTP. Structure from [74] (Protein Data Bank accession code 3IAY).
(PDF)

Figure S6 *eex* amino-acid substitutions in the exonuclease domain of Pol δ. The exo domain (red) is shown as a schematic of the α-carbon backbone, and exonuclease active-site residues are gray CPK sticks. Amino acids changed by *eex* mutations are shown as light blue spheres and labeled to indicate the *eex* substitutions. The red dotted line corresponds to a missing loop in the structure (amino acids 491–496). The β-hairpin in T4 and RB69 pols affects partitioning of the primer between polymerase and exonuclease active sites [4]. Structure from [74] (Protein Data Bank accession code 3IAY).
(PDF)

Table S1 Genotypes of candidate *eex* mutants.
(PDF)

Table S2 Types of spontaneous *CAN1* mutations in *pol3-01,eex msh6Δ* strains.
(PDF)

Table S3 Yeast strains.
(PDF)

Table S4 Construction of chromosomal gene disruptions.
(PDF)

Text S1 Supplementary methods.
(PDF)

## References

[1] Friedberg EC, Walker GC, Siede W, Wood RD, Schultz RA (2006). DNA Repair and Mutagenesis..

[2] McCulloch SD, Kunkel TA (2008). The fidelity of DNA synthesis by eukaryotic replicative and translesion synthesis polymerases.. Cell Res.

[3] Iyer RR, Pluciennik A, Burdett V, Modrich PL (2006). DNA mismatch repair: functions and mechanisms.. Chem Rev.

[4] Reha-Krantz LJ (2010). DNA polymerase proofreading: Multiple roles maintain genome stability.. Biochim Biophys Acta.

[5] de Visser JA (2002). The fate of microbial mutators.. Microbiology.

[6] Giraud A, Radman M, Matic I, Taddei F (2001). The rise and fall of mutator bacteria.. Curr Opin Microbiol.

[7] Sturtevant AH (1937). Essays on evolution. I. On the effects of selection on mutation rate.. Q Rev Biol.

[8] Denamur E, Matic I (2006). Evolution of mutation rates in bacteria.. Mol Microbiol.

[9] Drake JW, Charlesworth B, Charlesworth D, Crow JF (1998). Rates of spontaneous mutation.. Genetics.

[10] Elena SF, Lenski RE (2003). Evolution experiments with microorganisms: the dynamics and genetic bases of adaptation.. Nat Rev Genet.

[11] Chao L, Cox EC (1983). Competition between high and low mutating strains of *Escherichia coli*.. Evolution.

[12] Sniegowski PD, Gerrish PJ, Lenski RE (1997). Evolution of high mutation rates in experimental populations of *E. coli*.. Nature.

[13] Mao EF, Lane L, Lee J, Miller JH (1997). Proliferation of mutators in a cell population.. J Bacteriol.

[14] Giraud A, Matic I, Tenaillon O, Clara A, Radman M (2001). Costs and benefits of high mutation rates: adaptive evolution of bacteria in the mouse gut.. Science.

[15] Nilsson AI, Kugelberg E, Berg OG, Andersson DI (2004). Experimental adaptation of *Salmonella typhimurium* to mice.. Genetics.

[16] Notley-McRobb L, Seeto S, Ferenci T (2002). Enrichment and elimination of mutY mutators in Escherichia coli populations.. Genetics.

[17] Funchain P, Yeung A, Stewart JL, Lin R, Slupska MM (2000). The consequences of growth of a mutator strain of *Escherichia coli* as measured by loss of function among multiple gene targets and loss of fitness.. Genetics.

[18] Tröbner W, Piechocki R (1984). Selection against hypermutability in *Escherichia coli* during long term evolution.. Mol Gen Genet.

[19] Schaaper RM, Cornacchio R (1992). An *Escherichia coli dnaE* mutation with suppressor activity toward mutator *mutD5*.. J Bacteriol.

[20] Fijalkowska IJ, Schaaper RM (1995). Effects of *Escherichia coli dnaE* antimutator alleles in a proofreading-deficient *mutD5* strain.. J Bacteriol.

[21] Loeb LA, Springgate CF, Battula N (1974). Errors in DNA replication as a basis of malignant changes.. Cancer Res.

[22] Loeb LA, Bielas JH, Beckman RA (2008). Cancers exhibit a mutator phenotype: clinical implications.. Cancer Res.

[23] Bielas JH, Loeb KR, Rubin BP, True LD, Loeb LA (2006). Human cancers express a mutator phenotype.. Proc Natl Acad Sci USA.

[24] Goldsby RE, Lawrence NA, Hays LE, Olmsted EA, Chen X (2001). Defective DNA polymerase-δ proofreading causes cancer susceptibility in mice.. Nat Med.

[25] Albertson TM, Ogawa M, Bugni JM, Hays LE, Chen Y (2009). DNA polymerase ε and δ proofreading suppress discrete mutator and cancer phenotypes in mice.. Proc Natl Acad Sci USA.

[26] Goldsby RE, Hays LE, Chen X, Olmsted EA, Slayton WB (2002). High incidence of epithelial cancers in mice deficient for DNA polymerase δ proofreading.. Proc Natl Acad Sci USA.

[27] Wei K, Kucherlapati R, Edelmann W (2002). Mouse models for human DNA mismatch-repair gene defects.. Trends Mol Med.

[28] Peltomäki P (2005). Lynch syndrome genes.. Fam Cancer.

[29] Simon M, Giot L, Faye G (1991). The 3′ to 5′ exonuclease activity located in the DNA polymerase δ subunit of *Saccharomyces cerevisiae* is required for accurate replication.. EMBO J.

[30] Morrison A, Bell JB, Kunkel TA, Sugino A (1991). Eukaryotic DNA polymerase amino acid sequence required for 3′ → 5′ exonuclease activity.. Proc Natl Acad Sci USA.

[31] Williamson MS, Game JC, Fogel S (1985). Meiotic gene conversion mutants in *Saccharomyces cerevisiae*. I. Isolation and characterization of *pms1-1* and *pms1-2*.. Genetics.

[32] Reenan RA, Kolodner RD (1992). Characterization of insertion mutations in the *Saccharomyces cerevisiae MSH1* and *MSH2* genes: evidence for separate mitochondrial and nuclear functions.. Genetics.

[33] Prolla TA, Christie DM, Liskay RM (1994). Dual requirement in yeast DNA mismatch repair for *MLH1* and *PMS1*, two homologs of the bacterial *mutL* gene.. Mol Cell Biol.

[34] Strand M, Prolla TA, Liskay RM, Petes TD (1993). Destabilization of tracts of simple repetitive DNA in yeast by mutations affecting DNA mismatch repair.. Nature.

[35] Desai MM, Fisher DS, Murray AW (2007). The speed of evolution and maintenance of variation in asexual populations.. Curr Biol.

[36] Thompson DA, Desai MM, Murray AW (2006). Ploidy controls the success of mutators and nature of mutations during budding yeast evolution.. Curr Biol.

[37] Wloch DM, Szafraniec K, Borts RH, Korona R (2001). Direct estimate of the mutation rate and the distribution of fitness effects in the yeast *Saccharomyces cerevisiae*.. Genetics.

[38] Zeyl C, de Visser JAGM (2001). Estimates of the rate and distribution of fitness effects of spontaneous mutation in *Saccharomyces cerevisiae*.. Genetics.

[39] Zeyl C, Mizesko M, de Visser JAGM (2001). Mutational meltdown in laboratory yeast populations.. Evolution.

[40] Morrison A, Johnson AL, Johnston LH, Sugino A (1993). Pathway correcting DNA replication errors in *Saccharomyces cerevisiae*.. EMBO J.

[41] Tran HT, Gordenin DA, Resnick MA (1999). The 3′→5′ exonucleases of DNA polymerases δ and ε and the 5′→3′ exonuclease Exo1 have major roles in postreplication mutation avoidance in *Saccharomyces cerevisiae*.. Mol Cell Biol.

[42] Greene CN, Jinks-Robertson S (2001). Spontaneous frameshift mutations in *Saccharomyces cerevisiae*: accumulation during DNA replication and removal by proofreading and mismatch repair activities.. Genetics.

[43] Boeke JD, LaCroute F, Fink GR (1984). A positive selection for mutants lacking orotidine-5′-phosphate decarboxylase activity in yeast: 5-fluoro-orotic acid resistance.. Mol Gen Genet.

[44] Bernad A, Blanco L, Lazaro JM, Martin G, Salas M (1989). A conserved 3′→5′ exonuclease active site in prokaryotic and eukaryotic DNA polymerases.. Cell.

[45] Shevelev IV, Hübscher U (2002). The 3′–5′ exonucleases.. Nat Rev Mol Cell Biol.

[46] Morrison A, Sugino A (1994). The 3′→5′ exonucleases of both DNA polymerases δ and ε participate in correcting errors of DNA replication in *Saccharomyces cerevisiae*.. Mol Gen Genet.

[47] Marsischky GT, Filosi N, Kane MF, Kolodner R (1996). Redundancy of *Saccharomyces cerevisiae MSH3* and *MSH6* in *MSH2*-dependent mismatch repair.. Genes Dev.

[48] Johnson RE, Kovvali GK, Prakash L, Prakash S (1996). Requirement of the yeast *MSH3* and *MSH6* genes for *MSH2*-dependent genomic stability.. J Biol Chem.

[49] Alani E (1996). The Saccharomyces cerevisiae Msh2 and Msh6 proteins form a complex that specifically binds to duplex oligonucleotides containing mismatched DNA base pairs.. Mol Cell Biol.

[50] Sokolsky T, Alani E (2000). *EXO1* and *MSH6* are high-copy suppressors of conditional mutations in the *MSH2* mismatch repair gene of *Saccharomyces cerevisiae*.. Genetics.

[51] Hawk JD, Stefanovic L, Boyer JC, Petes TD, Farber RA (2005). Variation in efficiency of DNA mismatch repair at different sites in the yeast genome.. Proc Natl Acad Sci USA.

[52] Jin YH, Garg P, Stith CM, Al-Refai H, Sterling JF (2005). The multiple biological roles of the 3′→5′ exonuclease of *Saccharomyces cerevisiae* DNA polymerase δ require switching between the polymerase and exonuclease domains.. Mol Cell Biol.

[53] Schaaper RM (1993). Base selection, proofreading, and mismatch repair during DNA replication in *Escherichia coli*.. J Biol Chem.

[54] Pavlov YI, Shcherbakova PV, Rogozin IB (2006). Roles of DNA polymerases in replication, repair, and recombination in eukaryotes.. Int Rev Cytol.

[55] Giaever G, Chu AM, Ni L, Connelly C, Riles L (2002). Functional profiling of the *Saccharomyces cerevisiae* genome.. Nature.

[56] Winzeler EA, Shoemaker DD, Astromoff A, Liang H, Anderson K (1999). Functional characterization of the *S. cerevisiae* genome by gene deletion and parallel analysis.. Science.

[57] Goodman MF, Creighton S, Bloom LB, Petruska J (1993). Biochemical basis of DNA replication fidelity.. Crit Rev Biochem Mol Biol.

[58] Datta A, Schmeits JL, Amin NS, Lau PJ, Myung K (2000). Checkpoint-dependent activation of mutagenic repair in Saccharomyces cerevisiae pol3-01 mutants.. Mol Cell.

[59] Yu L, Pena Castillo L, Mnaimneh S, Hughes TR, Brown GW (2006). A survey of essential gene function in the yeast cell division cycle.. Mol Biol Cell.

[60] Ooi SL, Pan X, Peyser BD, Ye P, Meluh PB (2006). Global synthetic-lethality analysis and yeast functional profiling.. Trends Genet.

[61] Boone C, Bussey H, Andrews BJ (2007). Exploring genetic interactions and networks with yeast.. Nat Rev Genet.

[62] Fijalkowska IJ, Schaaper RM (1996). Mutants in the Exo I motif of *Escherichia coli dnaQ*: defective proofreading and inviability due to error catastrophe.. Proc Natl Acad Sci USA.

[63] Sliwa P, Kluz J, Korona R (2004). Mutational load and the transition between diploidy and haploidy in experimental populations of the yeast Saccharomyces cerevisiae.. Genetica.

[64] Delneri D, Hoyle DC, Gkargkas K, Cross EJM, Rash B (2008). Identification and characterization of high-flux-control genes of yeast through competition analyses in continuous cultures.. Nat Genet.

[65] Daee DL, Mertz TM, Shcherbakova PV (2010). A cancer-associated DNA polymerase δ variant modeled in yeast causes a catastrophic increase in genomic instability.. Proc Natl Acad Sci USA.

[66] Treuting PM, Albertson TM, Preston BD (2010). Case series: acute tumor lysis syndrome in mutator mice with disseminated lymphoblastic lymphoma.. Toxicol Pathol.

[67] Szafraniec K, Borts RH, Korona R (2001). Environmental stress and mutational load in diploid strains of the yeast Saccharomyces cerevisiae.. Proc Natl Acad Sci USA.

[68] Hillenmeyer ME, Fung E, Wildenhain J, Pierce SE, Hoon S (2008). The chemical genomic portrait of yeast: uncovering a phenotype for all genes.. Science.

[69] Gu Z, Steinmetz LM, Gu X, Scharfe C, Davis RW (2003). Role of duplicate genes in genetic robustness against null mutations.. Nature.

[70] Kafri R, Levy M, Pilpel Y (2006). The regulatory utilization of genetic redundancy through responsive backup circuits.. Proc Natl Acad Sci USA.

[71] Harrison R, Papp B, Pal C, Oliver SG, Delneri D (2007). Plasticity of genetic interactions in metabolic networks of yeast.. Proc Natl Acad Sci USA.

[72] Wagner A (2000). Robustness against mutations in genetic networks of yeast.. Nat Genet.

[73] Rutherford SL, Lindquist S (1998). Hsp90 as a capacitor for morphological evolution.. Nature.

[74] Swan MK, Johnson RE, Prakash L, Prakash S, Aggarwal AK (2009). Structural basis of high-fidelity DNA synthesis by yeast DNA polymerase δ.. Nat Struct Mol Biol.

[75] Albertson TM, Preston BD (2006). DNA replication fidelity: proofreading in trans.. Curr Biol.

[76] Trujillo KM, Sung P (2001). DNA structure-specific nuclease activities in the *Saccharomyces cerevisiae* Rad50·Mre11 complex.. J Biol Chem.

[77] Unk I, Haracska L, Prakash S, Prakash L (2001). 3′-Phosphodiesterase and 3′→5′ exonuclease activities of yeast Apn2 protein and requirement of these activities for repair of oxidative DNA damage.. Mol Cell Biol.

[78] Bardwell AJ, Bardwell L, Tomkinson AE, Friedberg EC (1994). Specific cleavage of model recombination and repair intermediates by the yeast Rad1-Rad10 DNA endonuclease.. Science.

[79] Bastin-Shanower SA, Fricke WM, Mullen JR, Brill SJ (2003). The mechanism of Mus81-Mms4 cleavage site selection distinguishes it from the homologous endonuclease Rad1-Rad10.. Mol Cell Biol.

[80] Boddy MN, Gaillard PH, McDonald WH, Shanahan P, Yates JR, 3rd (2001). Mus81-Eme1 are essential components of a Holliday junction resolvase.. Cell.

[81] Chen XB, Melchionna R, Denis CM, Gaillard PH, Blasina A (2001). Human Mus81-associated endonuclease cleaves Holliday junctions in vitro.. Mol Cell.

[82] Kaliraman V, Mullen JR, Fricke WM, Bastin-Shanower SA, Brill SJ (2001). Functional overlap between Sgs1-Top3 and the Mms4-Mus81 endonuclease.. Genes Dev.

[83] Drake JW, Allen EF, Forsberg SA, Preparata RM, Greening EO (1969). Genetic control of mutation rates in bacteriophage T4.. Nature.

[84] Reha-Krantz LJ (1988). Amino acid changes coded by bacteriophage T4 DNA polymerase mutator mutants. Relating structure to function.. J Mol Biol.

[85] Reha-Krantz LJ (1995). Use of genetic analyses to probe structure, function, and dynamics of bacteriophage T4 DNA polymerase.. Methods Enzymol.

[86] Reha-Krantz LJ, Wong C (1996). Selection of bacteriophage T4 antimutator DNA polymerases: a link between proofreading and sensitivity to phosphonoacetic acid.. Mutat Res.

[87] Hwang YT, Zuccola HJ, Lu Q, Hwang CB (2004). A point mutation within conserved region VI of herpes simplex virus type 1 DNA polymerase confers altered drug sensitivity and enhances replication fidelity.. J Virol.

[88] Hall JD, Coen DM, Fisher BL, Weisslitz M, Randall S (1984). Generation of genetic diversity in herpes simplex virus: an antimutator phenotype maps to the DNA polymerase locus.. Virology.

[89] Gibbs JS, Chiou HC, Bastow KF, Cheng YC, Coen DM (1988). Identification of amino acids in herpes simplex virus DNA polymerase involved in substrate and drug recognition.. Proc Natl Acad Sci USA.

[90] Loh E, Choe J, Loeb LA (2007). Highly tolerated amino acid substitutions increase the fidelity of *Escherichia coli* DNA polymerase I. J Biol Chem.

[91] Fijalkowska IJ, Dunn RL, Schaaper RM (1993). Mutants of *Escherichia coli* with increased fidelity of DNA replication.. Genetics.

[92] Schaaper RM (1996). Suppressors of *Escherichia coli mutT*: antimutators for DNA replication errors.. Mutat Res.

[93] Jin YH, Obert R, Burgers PM, Kunkel TA, Resnick MA (2001). The 3′→5′ exonuclease of DNA polymerase δ can substitute for the 5′ flap endonuclease Rad27/Fen1 in processing Okazaki fragments and preventing genome instability.. Proc Natl Acad Sci USA.

[94] Garg P, Stith CM, Sabouri N, Johansson E, Burgers PM (2004). Idling by DNA polymerase δ maintains a ligatable nick during lagging-strand DNA replication.. Genes Dev.

[95] Stith CM, Sterling J, Resnick MA, Gordenin DA, Burgers PM (2008). Flexibility of eukaryotic Okazaki fragment maturation through regulated strand displacement synthesis.. J Biol Chem.

[96] Pavlov YI, Maki S, Maki H, Kunkel TA (2004). Evidence for interplay among yeast replicative DNA polymerases alpha, delta and epsilon from studies of exonuclease and polymerase active site mutations.. BMC Biol.

[97] Preston BD, Albertson TM, Herr AJ (2010). DNA replication fidelity and cancer.. Semin Cancer Biol.

[98] Sherman F, Guthrie C, Fink GR (2002). Getting started with yeast.. Part B: Guide to Yeast Genetics and Molecular and Cell Biology.

[99] Toyn JH, Gunyuzlu PL, White WH, Thompson LA, Hollis GF (2000). A counterselection for the tryptophan pathway in yeast: 5-fluoroanthranilic acid resistance.. Yeast.

[100] Rose MD, Novick P, Thomas JH, Botstein D, Fink GR (1987). A *Saccharomyces cerevisiae* genomic plasmid bank based on a centromere-containing shuttle vector.. Gene.

[101] Giot L, Simon M, Dubois C, Faye G (1995). Suppressors of thermosensitive mutations in the DNA polymerase δ gene of *Saccharomyces cerevisiae*.. Mol Gen Genet.

[102] Gietz RD, Sugino A (1988). New yeast-*Escherichia coli* shuttle vectors constructed with in vitro mutagenized yeast genes lacking six-base pair restriction sites.. Gene.

[103] Brachmann CB, Davies A, Cost GJ, Caputo E, Li J (1998). Designer deletion strains derived from *Saccharomyces cerevisiae* S288C: a useful set of strains and plasmids for PCR-mediated gene disruption and other applications.. Yeast.

[104] Wach A, Brachat A, Pohlmann R, Philippsen P (1994). New heterologous modules for classical or PCR-based gene disruptions in *Saccharomyces cerevisiae*.. Yeast.

[105] Venkatesan RN, Hsu JJ, Lawrence NA, Preston BD, Loeb LA (2005). Mutator phenotypes caused by substitution at a conserved motif A residue in eukaryotic DNA polymerase δ.. J Biol Chem.

[106] Guldener U, Heck S, Fielder T, Beinhauer J, Hegemann JH (1996). A new efficient gene disruption cassette for repeated use in budding yeast.. Nucleic Acids Res.

[107] Pavlov YI, Newlon CS, Kunkel TA (2002). Yeast origins establish a strand bias for replicational mutagenesis.. Mol Cell.

[108] Lang GI, Murray AW (2008). Estimating the per-base-pair mutation rate in the yeast *Saccharomyces cerevisiae*.. Genetics.

[109] Rosche WA, Foster PL (2000). Determining mutation rates in bacterial populations.. Methods.

[110] Zheng Q (2002). Statistical and algorithmic methods for fluctuation analysis with SALVADOR as an implementation.. Math Biosci.

[111] Morgan C, Lewis PD (2006). iMARS--mutation analysis reporting software: an analysis of spontaneous cII mutation spectra.. Mutat Res.

[112] Drake JW (1991). A constant rate of spontaneous mutation in DNA-based microbes.. Proc Natl Acad Sci USA.

[113] Grogan DW, Carver GT, Drake JW (2001). Genetic fidelity under harsh conditions: analysis of spontaneous mutation in the thermoacidophilic archaeon Sulfolobus acidocaldarius.. Proc Natl Acad Sci USA.

[114] Drake JW (2009). Avoiding dangerous missense: thermophiles display especially low mutation rates.. PLoS Genet.

[115] Croyle ML, Woo AL, Lingrel JB (1997). Extensive random mutagenesis analysis of the Na+/K+-ATPase alpha subunit identifies known and previously unidentified amino acid residues that alter ouabain sensitivity--implications for ouabain binding.. Eur J Biochem.

[116] Fallows D, Kent RB, Nelson DL, Emanuel JR, Levenson R (1987). Chromosome-mediated transfer of the murine Na,K-ATPase alpha subunit confers ouabain resistance.. Mol Cell Biol.

[117] Price EM, Lingrel JB (1988). Structure-function relationships in the Na,K-ATPase alpha subunit: site-directed mutagenesis of glutamine-111 to arginine and asparagine-122 to aspartic acid generates a ouabain-resistant enzyme.. Biochemistry.

[118] Cantley LG, Cunha MJ, Zhou XM (1994). Ouabain-resistant OR6 cells express the murine alpha 1-subunit of the Na,K-ATPase with a T797-I797 substitution.. J Biol Chem.

[119] Dhillon KK, Sidorova JM, Albertson TM, Anderson JB, Ladiges WC (2010). Divergent cellular phenotypes of human and mouse cells lacking the Werner syndrome RecQ helicase.. DNA Repair.

[120] Wang TS-F, Wong SW, Korn D (1989). Human DNA polymerase α: predicted functional domains and relationships with viral DNA polymerases.. FASEB J.

[121] Tran HT, Degtyareva NP, Gordenin DA, Resnick MA (1999). Genetic factors affecting the impact of DNA polymerase δ proofreading activity on mutation avoidance in yeast.. Genetics.

[122] Hadjimarcou MI, Kokoska RJ, Petes TD, Reha-Krantz LJ (2001). Identification of a mutant DNA polymerase δ in *Saccharomyces cerevisiae* with an antimutator phenotype for frameshift mutations.. Genetics.

[123] Tian W, Hwang YT, Lu Q, Hwang CBC (2009). Finger domain mutation affects enzyme activity, DNA replication efficiency, and fidelity of an exonuclease-deficient DNA polymerase of herpes simplex virus type 1.. J Virol.

[124] Lynch M, Sung W, Morris K, Coffey N, Landry CR (2008). A genome-wide view of the spectrum of spontaneous mutations in yeast.. Proc Natl Acad Sci USA.

