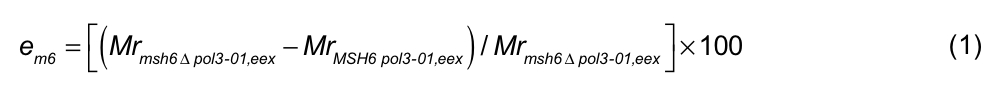# Correction: Mutator Suppression and Escape from Replication Error–Induced Extinction in Yeast

**DOI:** 10.1371/annotation/db1d9553-4ebd-4015-a1cd-c483dbc0d7e5

**Published:** 2011-11-02

**Authors:** Alan J. Herr, Masanori Ogawa, Nicole A. Lawrence, Lindsey N. Williams, Julie M. Eggington, Mallika Singh, Robert A. Smith, Bradley D. Preston

Equation 1 contains incorrect characters. Please view the correct equation here: