# Supplementary figures and images for: Mutator Suppression and Escape from Replication Error–Induced Extinction in Yeast
Source: PLoS Genet. 2011 Oct 6;7(10):e1002282. doi: 10.1371/journal.pgen.1002282 (PMC3188538; doi:10.1371/journal.pgen.1002282)

Figure S1  
Herr *et al.*

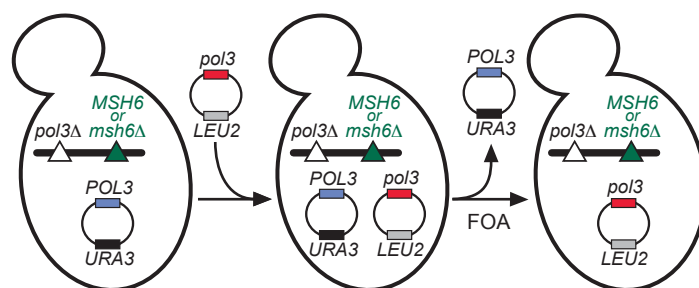

Supplement: Figure S1 — Plasmid shuffling strategy. Mutated pol3 alleles were introduced into MSH6 and msh6Δ yeast by plasmid shuffling. Haploid yeast with a chromosomal deletion of POL3 (pol3Δ) complemented by a wild-type POL3–URA3 plasmid (left) are transformed with mutant pol3–LEU2 plasmids. Individual colonies carrying both the pol3 and POL3 plasmids are isolated (center), and dispersed cells are then plated on 5-fluoroorotic acid (FOA) media to select mutant pol3–LEU2 clones that lost the wild-type POL3–URA3 vector (right). (PDF) [file pgen.1002282.s001.pdf]

Figure S4  
Herr *et al.*

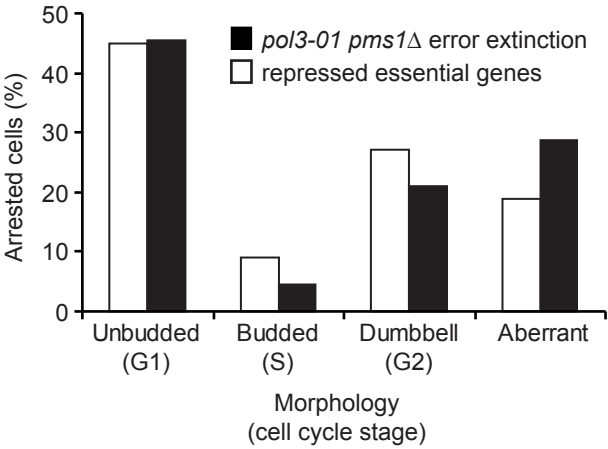

Supplement: Figure S4 — Similar cell morphologies in yeast after error extinction or repression of essential genes. Black bars: terminal cell morphologies of pol3-01 pms1Δ haploid cells that ceased growing due to error extinction [40]. White bars: cell-cycle arrest phenotypes of 563 haploid strains, each with a different repressed essential gene [59]. In the repression study, 82 additional essential genes showed a growth defect but with no defined cell-cycle arrest phenotype. (PDF) [file pgen.1002282.s004.pdf]

Figure S5  
Herr *et al.*

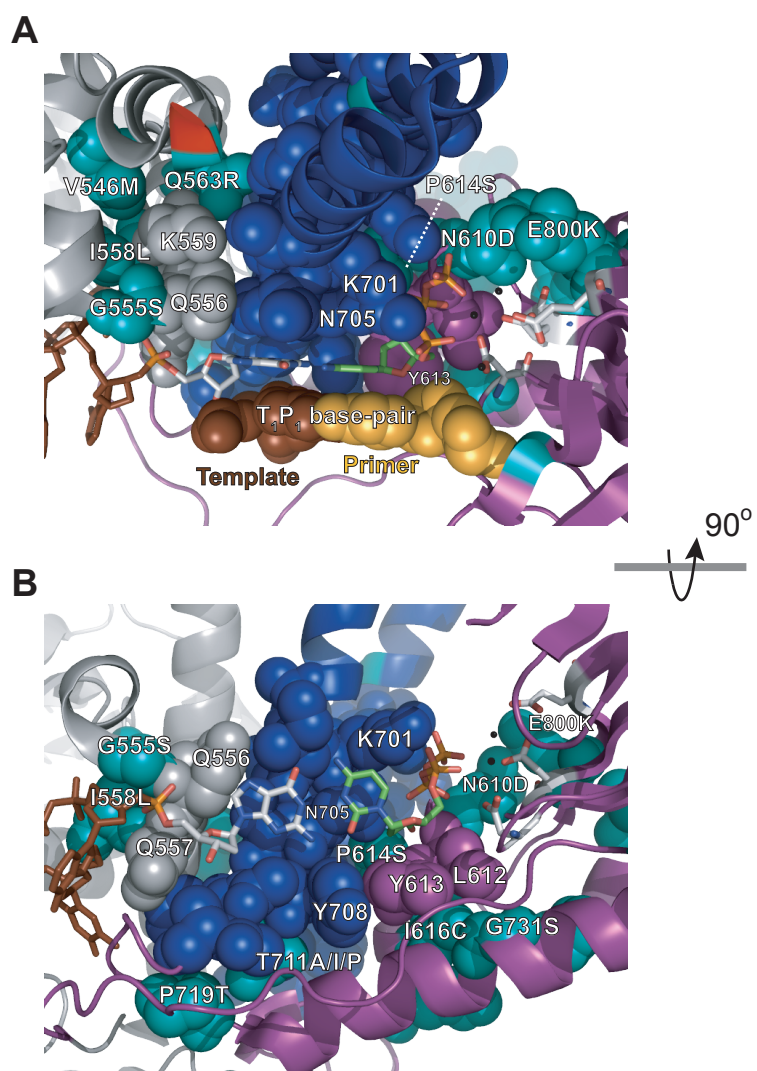

Supplement: Figure S5 — eex amino-acid substitutions near the template nucleotide in Pol δ. Schematic of the α-carbon backbone of Pol δ with residues of interest depicted as space-filling spheres. Structural elements are color-coded as in Figure 4 with the template•dNTP (T0P0) and polymerase active-site residues shown as CPK sticks. Amino acids changed by eex mutations are shown as light blue spheres and labeled to indicate the eex substitutions. Residues V546, G555, I558, and Q563 from the amino domain are in three closely associated α-helices that bind the template and buttress the fingers domain. The exo domain has been removed for clarity, and the penultimate T1P1 base-pair (brown and gold spheres) is included to delineate the binding pocket. Panel (A) is a view looking down on the DNA major groove. Panel (B) is the same image rotated 90° around the x-axis. The T1P1 base-pair was removed in Panel (B) to reveal positions of amino-acid substitutions around the template•dNTP. Structure from [74] (Protein Data Bank accession code 3IAY). (PDF) [file pgen.1002282.s005.pdf]

Figure S6  
Herr *et al.*

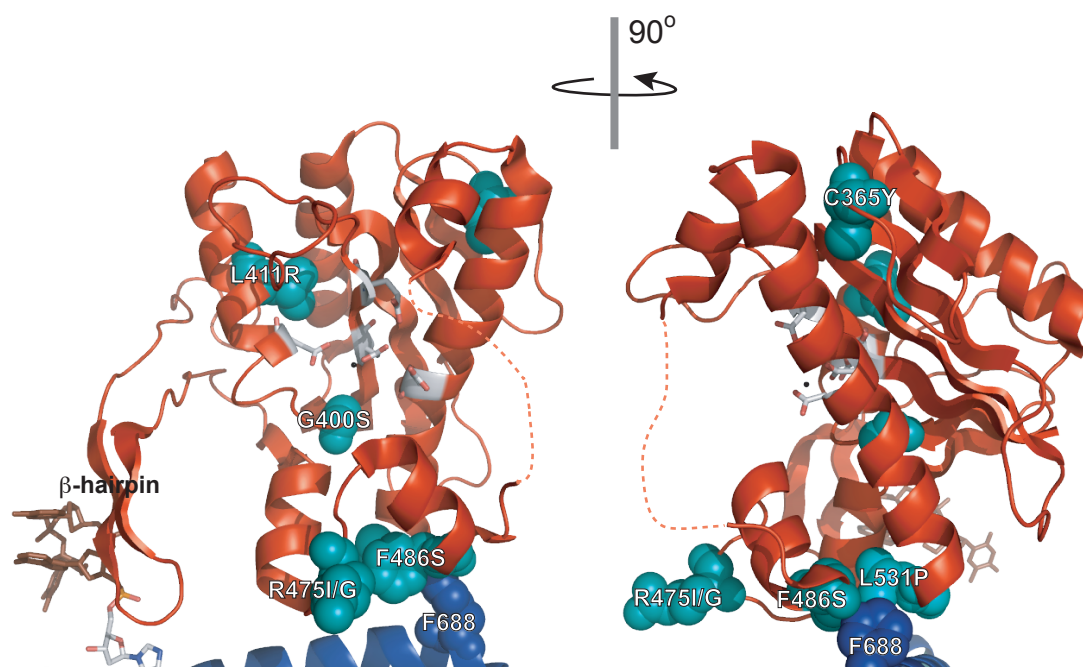

Supplement: Figure S6 — eex amino-acid substitutions in the exonuclease domain of Pol δ. The exo domain (red) is shown as a schematic of the α-carbon backbone, and exonuclease active-site residues are gray CPK sticks. Amino acids changed by eex mutations are shown as light blue spheres and labeled to indicate the eex substitutions. The red dotted line corresponds to a missing loop in the structure (amino acids 491–496). The β-hairpin in T4 and RB69 pols affects partitioning of the primer between polymerase and exonuclease active sites [4]. Structure from [74] (Protein Data Bank accession code 3IAY). (PDF) [file pgen.1002282.s006.pdf]
