# Supplementary material for: Mutator Suppression and Escape from Replication Error–Induced Extinction in Yeast
Source: PLoS Genet. 2011 Oct 6;7(10):e1002282. doi: 10.1371/journal.pgen.1002282 (PMC3188538; doi:10.1371/journal.pgen.1002282)
Supplement: Figure S2 — Mutagenesis screen of the Pol δ exonuclease domain. Each allele was engineered into a wild-type POL3 vector (YCplac111POL3) by site-directed mutagenesis and then introduced into yeast by plasmid shuffling (see Figure S1). Independent FOA-resistant colonies were patched onto synthetic complete (SC) plates and then replica-plated to SC plates lacking arginine and containing canavanine (60 µg/ml) to assess mutator phenotypes [47]. A) Representative canavanine plates used to assess mutator phenotypes. Each plate has twelve or thirteen patches of cells derived from independent colonies of the indicated genotypes. Spontaneous canavanine-resistant (Canr) mutants appear as small colonies in the ∼1-cm patches. Mutant frequencies were qualitatively judged from the lowest (wild type, WT) to highest (pol3-G400A msh6Δ) as indicated by –, +, ++ and +++ scores. B) Summary of POL3 alleles and corresponding mutator and growth phenotypes in MSH6 and msh6Δ cells. (PDF) [file pgen.1002282.s002.pdf]

A

Genotype

WT | *pol3-G400A* | *pol3-Y401A* | *pol3-G400A msh6Δ*

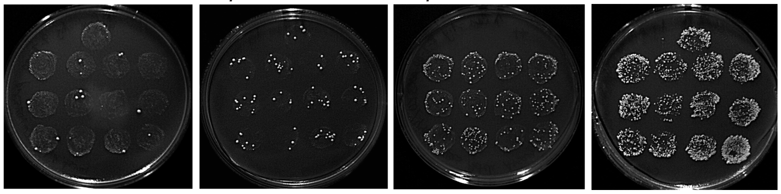

– | + | ++ | +++

Can^r Mutant Frequency

B

POL3 allele	Mutant Frequency[a] *MSH6*	Mutant Frequency[a] *msh6Δ*
Wild type	-	+
<u>Exo I motif</u>		
R316A	-	+
D321A	++	ND[b]
D321A,E323A[c]	++	no growth
G329A	-	+
<u>Exo II motif</u>		
P395A	-	+
D396A[d]	+	++/+++[e]
V397A	-	+
I398A	-	+
I399A	-	+
G400A	-/+	+++
Y401A	+/++	+++[e]
N402A	-	+
T403A	-	+
T404A	-	+
N405A	-	+
F406A	++	no growth
D407A/E/N/G[f]	++	no growth[g]
I408A	-	+
<u>Exo II motif</u> (continued)		
P409A	-	+
Y410A	-/+	++/+++
L411A	-	+
L412A	-	+
N413A	-	+
<u>Exo III motif</u>		
D506A/N[f,h]	-	+
S507A/N[f,h]	-	+
E508A/N[f,h]	-	+
R511A/H[f,h]	-	+
R512A	-	+
Y516F/T/S/I[f]	++	no growth[i]
D520A/T/V[f]	+/++	ND[e,j]
<u>Other</u>		
G447S	-	+
R459A[d]	-/+	+++
D463A	+/++	+++[e]
S476A	-	+
K491R	+	++/+++[e]

[a] Representative examples of –, +, ++ and +++ mutant frequencies are shown in panel A. Assayed in the isogenic *MSH6* and *msh6Δ* strains YP6 and MP4, respectively.
[b] ND, not determined.
[c] *pol3-01* allele (Morrison et al., 1993).
[d] Temperature-sensitive: normal growth at 30°C; no growth at 37°C.
[e] Slow growth.
[f] Slash (/) separates different individual *POL3* alleles assayed at this codon.
[g] Only the *D407A* allele was examined in *msh6Δ* cells.
[h] Mutator phenotypes assessed by fluctuation assays. - and + correspond to ≤3-fold and 10- to 20-fold increases in Can^r mutation rate relative to the wild-type strain, respectively. The mutation rates for wild type and *msh6Δ* were 2.0×10^{-7} and 2.5×10^{-6}, respectively, in these experiments.
[i] Only the *Y516F* allele was examined in *msh6Δ* cells.
[j] Only the *D520A* allele was examined for growth in *msh6Δ* cells.
