# Supplementary material for: Mutator Suppression and Escape from Replication Error–Induced Extinction in Yeast
Source: PLoS Genet. 2011 Oct 6;7(10):e1002282. doi: 10.1371/journal.pgen.1002282 (PMC3188538; doi:10.1371/journal.pgen.1002282)

Figure S3
Herr *et al.*

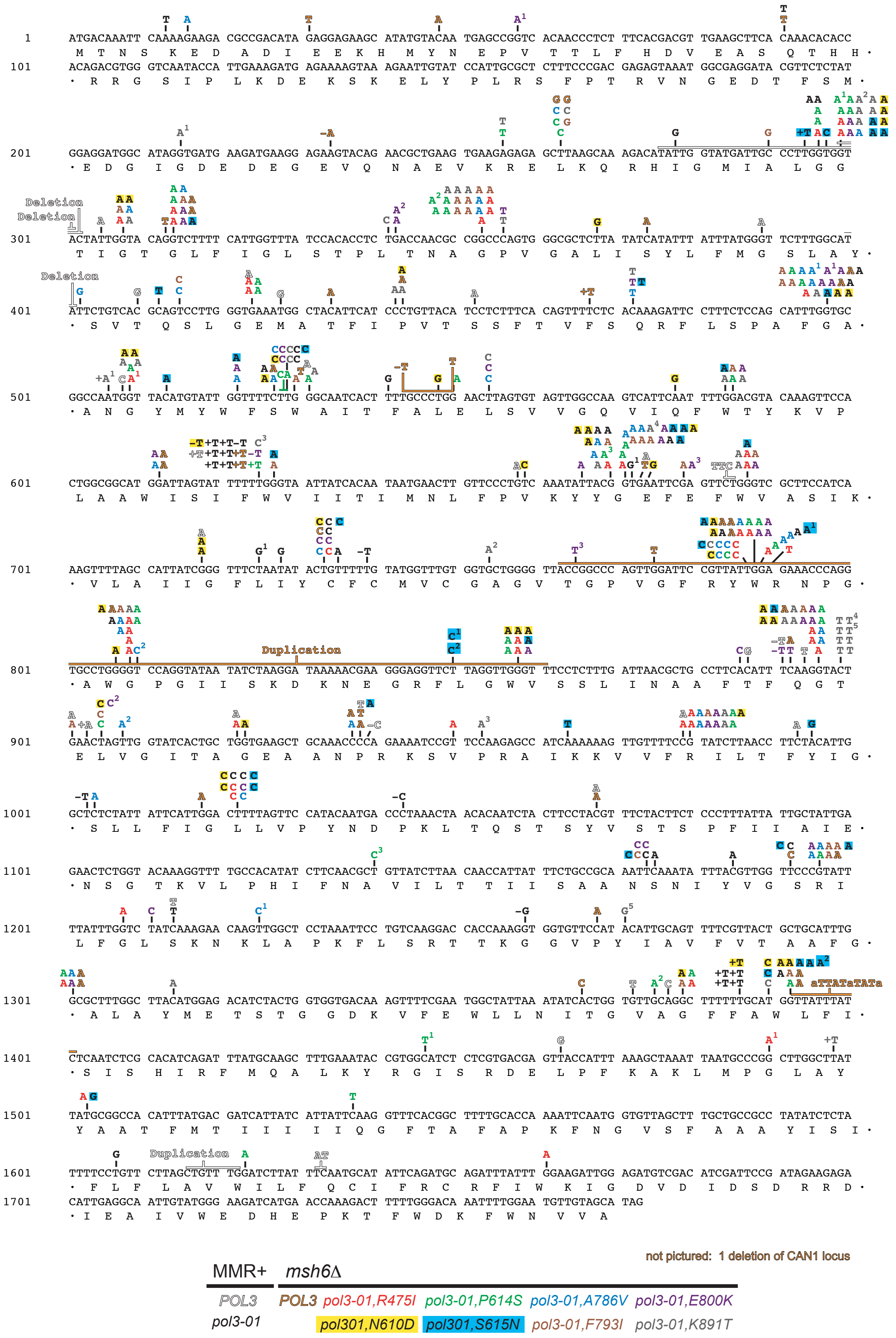

Supplement: Figure S3 — Spontaneous CAN1 mutations from pol3-01,eex msh6Δ cells. The can1 coding sequences from 30–48 independent canavanine-resistant (Canr) mutants of each strain were PCR-amplified and sequenced. Spontaneous mutations identified in different strains are color coded according to the key at the bottom. Each base letter above the wild-type CAN1 sequence indicates the site and nature of an independent base substitution or frameshift (+ or -) mutation. CAN1 sequences involved in complex mutations are indicated by horizontal colored lines. Multiple mutations identified in can1 from the same mutant clone are designated by the same superscript in the same color code. We observed mutation hotspots in CAN1 that arose in multiple independent Canr clones. One hotspot, a C to T mutation at nt 899, occurred in eight independent pol3-01,K891T Canr clones. Two of the eight mutants had a second mutation elsewhere in the CAN1 sequence, unambiguously identifying each clone as unique. This suggests that the abundance of mutations at this site is unlikely to be an artifact. One Canr POL3 msh6Δ mutant (not shown) contained an insertion/deletion mutation that was evident by a larger PCR product; sequencing with nested CAN1-specific primers revealed an insertion containing the gene RRP45. (PDF) [file pgen.1002282.s003.pdf]
