# Supplementary material for: Mutator Suppression and Escape from Replication Error–Induced Extinction in Yeast
Source: PLoS Genet. 2011 Oct 6;7(10):e1002282. doi: 10.1371/journal.pgen.1002282 (PMC3188538; doi:10.1371/journal.pgen.1002282)
Supplement: Table S1 — Genotypes of candidate eex mutants. (PDF) [file pgen.1002282.s007.pdf]

Table S1. Genotypes of Candidate *eex* Mutants

Class of FOA^r Mutant	# of Clones
eex	
pol3-01 intragenic	39
chromosomal	74
	113
ura3 mutation	352
pol3-01→POL3 conversion	4
Total	469

FOA^r colonies from 469 different *pol3-01–LEU POL3–URA msh6Δ* parent clones were isolated and genotyped to distinguish genuine *eex* mutants from *ura3* mutations and *pol3-01→POL3* gene conversions. Plasmids from *eex* mutants were recovered and retested in a fresh *msh6Δ* strain to distinguish second-site suppressors within *pol3-01* ('intragenic' *eex*) from *eex* mutations located on chromosomes in the original mutants ('chromosomal' *eex*). The 113 *eex* mutants include two *pol3-01* intragenic *eex* isolated in the initial experiment (Figure 2B).
