# Supplementary material for: Mutator Suppression and Escape from Replication Error–Induced Extinction in Yeast
Source: PLoS Genet. 2011 Oct 6;7(10):e1002282. doi: 10.1371/journal.pgen.1002282 (PMC3188538; doi:10.1371/journal.pgen.1002282)
Supplement: Table S2 — Types of spontaneous CAN1 mutations in pol3-01,eex msh6Δ strains. (PDF) [file pgen.1002282.s008.pdf]

Table S2. Types of Spontaneous *CAN1* Mutations in *pol3-01,eex msh6Δ* Strains [a]

				pol3-01,eex msh6Δ							
				Polymerase Active Site [b]						DNA Binding [b]	
	WT	*msh6Δ*	*pol3-01*	*N610D*	*P614S*	*S615N*	*A786V*	*F793I*	*E800K*	*R475I*	*K891T*
Transitions											
G→A	7 (22)	16 (34)	11 (23)	31 (66)	38 (79)	20 (51)	28 (62)	29 (85)	30 (63)	36 (78)	25 (49)
C→T	3 (9)	2 (4)	2 (4)	0 (0)	1 (2)	2 (5)	1 (2)	0 (0)	4 (8)	0 (0)	10 (20)
A→G	0 (0)	0 (0)	0 (0)	2 (4)	0 (0)	1 (3)	0 (0)	0 (0)	0 (0)	0 (0)	1 (2)
T→C	0 (0)	3 (6)	8 (17)	8 (17)	6 (13)	11 (28)	11 (24)	3 (9)	10 (21)	5 (11)	8 (16)
	10 (31)	21 (45)	21 (45)	41 (87)	45 (94)	34 (87)	40 (89)	32 (94)	44 (92)	41 (89)	44 (86)
Transversions											
A→C	1 (3)	1 (2)	0 (0)	0 (0)	0 (0)	0 (0)	0 (0)	0 (0)	1 (2)	0 (0)	0 (0)
A→T	1 (3)	0 (0)	1 (2)	0 (0)	1 (2)	1 (3)	0 (0)	0 (0)	0 (0)	1 (2)	1 (2)
C→A	3 (9)	10 (21)	3 (6)	2 (4)	0 (0)	2 (5)	3 (7)	1 (3)	0 (0)	1 (2)	4 (8)
C→G	2 (6)	0 (0)	0 (0)	0 (0)	0 (0)	0 (0)	0 (0)	0 (0)	0 (0)	0 (0)	0 (0)
G→C	1 (3)	0 (0)	0 (0)	0 (0)	0 (0)	0 (0)	0 (0)	0 (0)	0 (0)	0 (0)	0 (0)
G→T	2 (6)	5 (11)	0 (0)	0 (0)	0 (0)	0 (0)	0 (0)	0 (0)	0 (0)	0 (0)	0 (0)
T→A	1 (3)	0 (0)	1 (2)	0 (0)	0 (0)	0 (0)	1 (2)	0 (0)	1 (2)	3 (7)	0 (0)
T→G	1 (3)	2 (4)	6 (13)	2 (4)	0 (0)	1 (3)	1 (2)	1 (3)	0 (0)	0 (0)	0 (0)
	12 (38)	18 (38)	11 (23)	4 (9)	1 (2)	4 (10)	5 (11)	2 (6)	2 (4)	5 (11)	5 (10)
Frameshifts											
-1	2 (6)	1 (2)	5 (11)	1 (2)	0 (0)	0 (0)	0 (0)	0 (0)	2 (4)	0 (0)	0 (0)
+1	2 (6)	3 (6)	10 (21)	1 (2)	1 (2)	1 (3)	0 (0)	0 (0)	0 (0)	0 (0)	2 (4)
	4 (13)	4 (9)	15 (32)	2 (4)	1 (2)	1 (3)	0 (0)	0 (0)	2 (4)	0 (0)	2 (4)
Other											
	6 (19)	4 (9)	0 (0)	0 (0)	1 (2)	0 (0)	0 (0)	0 (0)	0 (0)	0 (0)	0 (0)
Multiple											
	0	0	1	0	3	2	2	0	3	1	5
Total											
	32	47	47	47	48	39	45	34	48	46	51

[a] The *CAN1* gene was sequenced from Can^r mutants of each strain. The numbers of mutations of each subtype are shown with percentages in parentheses. 'Other' includes duplications, deletions, and complex mutations. Some mutants had two mutations separated by more than 10 bp (61–1150 bp). These are reported under 'Multiple'; each mutation in this category was scored as an independent event and added to the relevant subclass tally, although they may be mechanistically linked. See Figure S3 for locations of mutations in the *CAN1* gene.

[b] Alleles are grouped based on the locations of affected amino acids in the Pol δ structure (see Figures 4, S5 and S6).
