# Supplementary material for: Mutator Suppression and Escape from Replication Error–Induced Extinction in Yeast
Source: PLoS Genet. 2011 Oct 6;7(10):e1002282. doi: 10.1371/journal.pgen.1002282 (PMC3188538; doi:10.1371/journal.pgen.1002282)
Supplement: Table S3 — Yeast strains. (PDF) [file pgen.1002282.s009.pdf]

Table S3. Yeast Strains

Strain	Relevant Genotype	Reference
YGL27-3D [a]	*pol3::HIS3* + pGL310 [*URA3/POL3*]	[29,101]
YGL27-3Dmsh6dis4	*pol3::HIS3 msh6::TRP1* + pGL310 [*URA3/POL3*]	This Study
YP6	*pol3::kanMX* + pGL310 [*URA3/POL3*]	This Study
MP4	*pol3::kanMX msh6::TRP1* + pGL310 [*URA3/POL3*]	This Study
BY4733 [b]		[103]
P3H3a	*pol3::HIS3* + pGL310 [*URA3/POL3*]	This Study
BP0109	*pol3::HIS3 msh2::TRP1* + pGL310 [*URA3/POL3*]	This Study
BP1506	*pol3::HIS3 msh6::kanMX* + pGL310 [*URA3/POL3*]	This Study
BP4001	*pol3::HIS3 agp1::URA3* + pRS414POL3 [*TRP1/POL3*]	This Study

[a] YGL27-3D, *MATα leu2 ura3 trp1 lys2 his3 ade2 CAN1 pol3::HIS3* + pGL310 [*URA3/POL3*]. YGL27-3D (kindly provided by Michel Simon and Gerard Faye, Institut Curie) harbors a lethal partial deletion of the chromosomal *POL3* gene substituted by a *HIS3* cassette; *POL3* function is provided by a wild-type copy of the gene with its natural promoter carried on the plasmid pGL310 [29,101]. pGL310 is the *CEN4/ARS1/URA3* plasmid YCp50 [100] modified to carry *SUP11* and *POL3* [29,101]. YP6 (previously called YGL27-*pol3Δ*; [105]) and MP4 were generated from YGL27-3D and YGL27-3Dmsh6dis4, respectively, by replacing *pol3::HIS3* in each strain with a *kanMX* cassette [104] that deletes the entire chromosomal *POL3* gene.

[b] BY4733, *MAT**a** leu2Δ0 ura3Δ0 met15Δ0 trp1Δ63 his3Δ200*. BY4733 is a S288C descendent [103] that we re-derived via sporulation of a BY4733 X BY4734 diploid (kindly provided by Tim Formosa, University of Utah). P3H3a and the BP series were constructed from this re-derived BY4733 strain by first introducing pGL310 or pRS414POL3 to provide a wild-type plasmid copy of *POL3* and then replacing the entire chromosomal *POL3* gene with a *HIS3* cassette. BP4001 has *URA3* inserted at the *agp1* locus near *ARS306* on chromosome III [Nick McElhinny et al. (2008) Mol Cell 30: 137-144]. pRS414POL3 is the *CEN6/ARSH4/TRP1* plasmid pRS414 [103] carrying wild-type *POL3* with its natural promoter.
