# Supplementary material for: Mutator Suppression and Escape from Replication Error–Induced Extinction in Yeast
Source: PLoS Genet. 2011 Oct 6;7(10):e1002282. doi: 10.1371/journal.pgen.1002282 (PMC3188538; doi:10.1371/journal.pgen.1002282)
Supplement: Table S4 — Construction of chromosomal gene disruptions. (PDF) [file pgen.1002282.s010.pdf]

Table S4. Construction of Chromosomal Gene Disruptions

Allele	PCR Primer Name	PCR Primer Sequence	PCR Template
msh6::TRP1 [a]	21355	5′-ATGGCCCCAGCTACCCCTAAAACTTC-3′	YGL27-3D genomic DNA and YIplac204 (Gietz and Sugino, 1988)
	21737	5′-CAGAGCAGATTGTACTGAGAGTGCACCATATTCGAGATTGGGAAGATGATAAATCCTC-3′	
	21738	5′-GATCCTTGAGAGTTTTCGCCCCGAAGAACGGAGGTGGACTCGAAAACGAATACTC-3′	
	21740	5′-GTTCCAATCATAGTTCAAGAC-3′	
pol3::kanMX [a]	POL3F	5′-ATATTGAGCACTTGCTATTAAGCATTAATCTTATACATATACGCACAGCACTGTTTAGCTTGCCTCGTCC-3′	pFA6-kanMX (Wach et al., 1994)
	POL3R	5′-TTGTTAGCCTTTCTTAATCCTAATATGATGTGCCACCCTATCGTTTTTTAGAATCGACAGCAGTATAGCG-3′	
pol3::HIS3 [b]	Pol3U	5′-ATATTGAGCACTTGCTATTAAGCATTAATCTTTATACATATACGCACAGCAAGATTGTACTGAGAGTGCAC-3′	pRS413 (Brachmann et al., 1998)
	Pol3D	5′-TTGTTAGCCTTTCTTAATCCTAATATGATGTGCCACCCTATCGTTTTTTACTGTGCGGTATTTCACACCG-3′	
msh6::kanMX [b]	kanmsh6U	5′-TTTAATTGGAGCAACTAGTTAATTTTGACAAAGCCAATTTGAACTCCAAAGAAGTTATTAGGTCTAGAGATCTG-3′	pUG6 (Guldener et al., 1996)
	kanmsh6D	5′-ACTTTAAAAAAAATAAGTAAAAATCTTACATACATCGTAAATGAAAATACACGAAGTTATATTAAGGGTTCTCG-3′	
msh2::TRP1 [b]	Msh2U	5′-AAAAATCTCTTTATCTGCTGACCTAACATCAAAATCCTCAGATTAAAAGTAGATTGTACTGAGAGTGCAC-3′	pRS414 (Brachmann et al., 1998)
	Msh2D	5′-TTATAACAACAAGGCTTTTATATATTTCAGGTAATTATCGTTTTCCTTTTCTGTGCGGTATTTCACACCG-3′	
agp1::URA3 [b]	AGP1URA3-R1F	5′-ATGTAATCTTTATAGAAGAAGCACGCTAATATAGACAAAGATAGCTTCGCACAAGATTGTACTGAGAGTGCAC-3′	pRS416 (Brachmann et al., 1998)
	AGP1URA3-R1R	5′-ACACCAGAAGGCAACGACCCTTTTCCAATAAGGTCCGTTCCTCAAACGTTCCCTGTGCGGTATTTCACACCG-3′	

Mutations were introduced into yeast using PCR products generated with the indicated primers and template DNAs. Underlined sequences are complementary to the PCR template plasmids 5′ and 3′ of each marker gene (*kanMX*, *HIS3*, *TRP1*, or *URA3*). Non-underlined sequences correspond to portions of each yeast gene targeted for homologous disruption (*POL3*, *MSH6*, *MSH2*, or *AGP1*). PCR amplification conditions are described in Text S1.

[a] Used to make disruptions in strains derived from YGL27-3D.

[b] Used to make disruptions in strains derived from BY4733.
