# Supplementary material for: Mutator Suppression and Escape from Replication Error–Induced Extinction in Yeast
Source: PLoS Genet. 2011 Oct 6;7(10):e1002282. doi: 10.1371/journal.pgen.1002282 (PMC3188538; doi:10.1371/journal.pgen.1002282)
Supplement: Text S1 — Supplementary methods. (PDF) [file pgen.1002282.s011.pdf]

Text S1. Supplementary Methods

DNA Cloning

Standard molecular biology procedures were employed for manipulating DNA and cloning in *E. coli* [1]. Plasmid DNA preparations were performed with QIAprep Spin (Qiagen) or Wizard (Promega) miniprep kits. Gel purifications of DNA fragments utilized the QIAquick Gel Extraction Kit (Qiagen). DNA transformations of yeast were achieved by a lithium acetate method [2]. DNA for Southern blot analyses was prepared as described [3].

Plasmid Construction and Mutagenesis

YCplac111*POL3* and mutant *pol3* variants for scanning mutagenesis (Figure S2) were generated as follows. First, the *POL3* gene was cloned in two portions to facilitate site-directed mutagenesis. The N-terminal region of *POL3* was PCR-amplified from yeast genomic DNA with primers H3aF (5′-TTTGCAGGGGACCTCTTGGAA-3′) and BHIR (5′-ACCGTCGCTCCTGTAAAA CCA-3′), and the major HindIII-BamHI fragment present in the PCR product was cloned into the corresponding sites of pAlter-1 (Promega). The resulting plasmid, pAlt1-5′*POL3*, contained the 2.5-kb N-terminal region of *POL3*, including the native promoter and conserved Exo I, II, and III motifs. The 1.5-kb C-terminal region of *POL3* was PCR-amplified from yeast genomic DNA with primers BHIF (5′-GGAAATGTTGAGCAATTGGCG-3′) and H3bR (5′-CAGTGATCAATTGGGGCCACC-3′), digested with BsaI, blunted with the Klenow fragment of *E. coli* Pol I, restricted by BamHI, and then cloned into the BamHI and SmaI sites in YCplac111 [4], creating YCplac111-3′*POL3*. The *pol3-01* mutations (encoding D321A and E323A amino-acid changes; [5]) and all single-base substitutions in the scanning mutagenesis experiment (Figure S2) were introduced into pAlt1-5′*POL3* by oligonucleotide-mediated site-directed mutagenesis according to the pAlter-1 manufacturer's protocol (Promega). Oligonucleotide sequences and PCR conditions are available upon request.

Sequence-verified wild-type and mutant pAlt1-5′*POL3* plasmids were restricted with HindIII and BamHI, gel purified, and ligated into the HindIII and BamHI sites of YCplac111-3′*POL3*. The ligations were transformed directly into yeast strains YP6 or MP4 (Table S3) followed by selection on SC media lacking leucine and uracil. Leu^+ Ura^+ colonies were plated on SC FOA media to select for loss of pGL310. The presence of each mutation and the absence of wild-type *POL3* in the mutant derivatives were confirmed by plasmid rescue in *E. coli* and sequencing of the entire *POL3* gene from each YCplac111*POL3* derivative. Our initial full-length *POL3* clones were unstable in *E. coli* at 37°C as observed with other *POL3*-bearing plasmids [6]; this instability was alleviated by propagation at 30°C, allowing us to perform subsequent manipulations of plasmids carrying full-length *POL3* directly in *E. coli* using the *recA1* strain, DH5α.

To create pRS414*POL3* and pRS414*pol3-01*, the respective HindIII-EcoRI fragments from YCplac111*POL3* and YCplac111*pol3-01*, encompassing the entire *POL3* regulatory and coding sequences, were cloned into the HindIII and EcoRI sites in the multiple cloning site of pRS414 [7]. The pRS414 vector contains a second HindIII site within the *TRP1* gene (closer to the first HindIII site than to the EcoRI site). Thus, a partial digestion with HindIII was utilized to prepare the vector. Ten *μ*g of pRS414 was first digested completely with EcoRI, then ten units of HindIII were added to a final volume of 60 *μ*l. Aliquots (15 *μ*l) were removed at four-minute intervals and quenched by the addition of 10 mM EDTA. The samples were resolved on a 0.75% agarose gel to monitor release of the 950-bp HindIII-HindIII fragment, and DNA from samples with only partial release of the HindIII fragment were used for cloning.

The *eex* mutations isolated in YCplac111*pol3,* YCplac111*pol3-01,* and pRS414*pol3-01* were re-engineered into fresh YCplac111*POL3,* YCplac111*pol3,* and YCplac111*pol3-01* plasmids as follows. Mutations that were 3′ of the Exo motifs were subcloned into the appropriate vector backbone using EagI-BamHI or BamHI-EcoRI fragments isolated from the

original mutant plasmids. A subset of *eex* mutations fell within a HindIII-EagI fragment that also included the *pol3-01* mutation. These *eex* alleles were re-engineered into fresh YCplac111 vectors by subcloning the HindIII-EagI fragments into YCplac111*POL3* (to generate *pol3-01,eex* plasmids) or by site-directed mutagenesis of YCplac111*POL3* (to generate *pol3-eex* plasmids). Site-directed mutagenesis was achieved using Stratagene's Quick-Change protocol (oligonucleotide sequences available on request). The resultant mutagenized HindIII-EagI fragments were then ligated into fresh YCplac111*POL3* vectors. All re-engineered mutations were confirmed by sequencing the cloned insert and flanking sequences prior to mutation rate and plating efficiency determinations.

Yeast Strain Construction

The YGL27-3Dmsh6dis4 strain (Table S3) was created by replacement of the *MSH6* gene in YGL27-3D with *TRP1*. A PCR strategy was used to generate the *TRP1* disruption cassette; the method and primer sequences (Table S4) were based on those used previously to disrupt *MSH6* [8]. Briefly, sequences from the 5′ and 3′ ends of *MSH6* were PCR-amplified with primers containing additional sequences that include the XmnI and NdeI sites flanking *TRP1* in the plasmid YIplac204. The 2.6-kb disruption fragment was created by PCR-amplifying these 0.4-kb 5′ and 0.9-kb 3′ *MSH6* fragments together with a XmnI/NdeI digest of YIplac204 using the two outside primers. This yielded a fragment containing *TRP1* flanked by sequences homologous to the 5´ and 3´ regions of *MSH6* in the yeast chromosome. The fragment was gel-purified and transformed into YGL27-3D. Transformants were selected on SC media lacking tryptophan, and the disruption verified by PCR and Southern blot analysis of independent colonies.

YP6 (previously call YGL27-*pol3Δ*; [9]) and MP4 were generated from YGL27-3D and YGL27-3Dmsh6dis4, respectively. The entire open reading frames of genomic *POL3* in the YGL27-3D and YGL27-3Dmsh6dis4 strains were deleted using a DNA fragment generated by PCR amplification of the *kanMX* module in pFA6-kanMX [10] using primers POL3F and POL3R

(Table S4). PCR products, containing 50 bp of homology to either end of the POL3 coding sequence, were purified (QIAquick PCR Clean-Up kit; Qiagen) and transformed into yeast, which were then plated on YPD media. After 24 hours, the yeast were replica-plated to YPD plates containing G418 (200 μg/ml) and incubated for 2-3 days. DNA was extracted from G418-resistant clones, and the *POL3* disruption was verified by PCR, Southern blot, and sequence analyses.

Deletion/replacement cassettes for modification of BY4733 (Table S3) were created by PCR-amplification of plasmid selectable markers using primers containing 50-nucleotide 5′ overhangs homologous to each end of the gene targeted for disruption (Table S4). The resultant PCR fragments were gel purified and transformed into yeast as described above. All chromosomal modifications were confirmed by genomic PCR using primers outside of the targeted region and internal primers within the replacement sequence (primer sequences available on request).

***POL3* and *pol3-01* Genotyping**

The *pol3-01* allele disrupts an EcoRV site, providing a convenient marker for genotyping. Cells treated with Zymolyase (ICN Biomedicals; 50 u/ml in 10 mM Tris•HCl/0.1 mM EDTA, pH7.5 at 37°C for 30 min, then 95°C for 10 min) were subjected to PCR amplification across the *pol3-01* mutant region in 20-μl reactions using primers Pol3GTF (5′-AGAGTTTCCTCTTGTCAGTTGGAAGTTTCAATTA-3′) and Pol3GTR (5′-CAGCAATACTCACAACGTTGGCAATT- 3′) with *Taq* polymerase (Qiagen) in the Qiagen PCR buffer with the following amplification conditions: 94°C, 2 min; 25 x (94°C, 20 sec; 54°C, 30 sec, 72°C, 30 sec); 72°C, 2 min. The samples were then digested with 10 units EcoRV for 1 hour and resolved on a 2.5% agarose gel in TBE. A single product of 196 bp is observed for *pol3-01*, while two fragments (111 and 85 bp) are observed for POL3. Strains containing both *POL3* and *pol3-01* yield all three fragments in this assay.

References

1. Sambrook J, Fritsch, E.F., Maniatis, T. (1989) Molecular Cloning: A Laboratory Manual. Cold Spring Harbor: Cold Spring Harbor Laboratory Press.

2. Gietz RD, Woods RA (2002) Transformation of yeast by lithium acetate/single-stranded carrier DNA/polyethylene glycol method. In: Guthrie C, Fink GR, editors. Part B: Guide to Yeast Genetics and Molecular and Cell Biology. Volume 350 ed. San Diego: Academic Press. pp. 87-96.

3. Hoffman CS, Winston F (1987) A ten-minute DNA preparation from yeast efficiently releases autonomous plasmids for transformation of *Escherichia coli*. Gene 57: 267-272.

4. Gietz RD, Sugino A (1988) New yeast-*Escherichia coli* shuttle vectors constructed with in vitro mutagenized yeast genes lacking six-base pair restriction sites. Gene 74: 527-534.

5. Morrison A, Johnson AL, Johnston LH, Sugino A (1993) Pathway correcting DNA replication errors in *Saccharomyces cerevisiae*. EMBO J 12: 1467-1473.

6. Simon M, Giot L, Faye G (1991) The 3´ to 5´ exonuclease activity located in the DNA polymerase δ subunit of *Saccharomyces cerevisiae* is required for accurate replication. EMBO J 10: 2165-2170.

7. Brachmann CB, Davies A, Cost GJ, Caputo E, Li J, et al. (1998) Designer deletion strains derived from *Saccharomyces cerevisiae* S288C: a useful set of strains and plasmids for PCR-mediated gene disruption and other applications. Yeast 14: 115-132.

8. Marsischky GT, Filosi N, Kane MF, Kolodner R (1996) Redundancy of *Saccharomyces cerevisiae MSH3* and *MSH6* in *MSH2*-dependent mismatch repair. Genes Dev 10: 407-420.

9. Venkatesan RN, Hsu JJ, Lawrence NA, Preston BD, Loeb LA (2005) Mutator phenotypes caused by substitution at a conserved motif A residue in eukaryotic DNA polymerase δ. J Biol Chem 281: 4486-4494.

10. Wach A, Brachat A, Pohlmann R, Philippsen P (1994) New heterologous modules for classical or PCR-based gene disruptions in *Saccharomyces cerevisiae*. Yeast 10: 1793-1808.
